# Physiology-Based Revascularization: A New Approach to Plan and Optimize Percutaneous Coronary Intervention

**DOI:** 10.1016/j.jacasi.2021.03.002

**Published:** 2021-05-21

**Authors:** Joo Myung Lee, Seung Hun Lee, Doosup Shin, Ki Hong Choi, Tim P. van de Hoef, Hyun Kuk Kim, Habib Samady, Tsunekazu Kakuta, Hitoshi Matsuo, Bon-Kwon Koo, William F. Fearon, Javier Escaned

**Affiliations:** a Division of Cardiology, Department of Internal Medicine, Heart Vascular Stroke Institute, Samsung Medical Center, Sungkyunkwan University School of Medicine, Seoul, Republic of Korea; b Division of Cardiovascular Medicine, Department of Internal Medicine, University of Iowa Carver College of Medicine, Iowa City, Iowa, USA; c Department of Clinical and Experimental Cardiology, Amsterdam UMC–University of Amsterdam, Amsterdam, the Netherlands; d Department of Internal Medicine and Cardiovascular Center, Chosun University Hospital, University of Chosun College of Medicine, Gwangju, Republic of Korea; e Andreas Gruentzig Cardiovascular Center, Emory University School of Medicine, Atlanta, Georgia, USA; f Division of Cardiovascular Medicine, Tsuchiura Kyodo General Hospital, Ibaraki, Japan; g Department of Cardiovascular Medicine, Gifu Heart Center, Gifu, Japan; h Department of Internal Medicine and Cardiovascular Center, Seoul National University Hospital, Seoul, Republic of Korea; i Division of Cardiovascular Medicine, Stanford Cardiovascular Institute, Stanford University School of Medicine, Stanford, California, USA; j Hospital Clínico San Carlos, IDISSC, and Universidad Complutense de Madrid, Madrid, Spain

**Keywords:** fractional flow reserve, instantaneous wave-free ratio, nonhyperemic pressure ratios, percutaneous coronary intervention, prognosis, CI, confidence interval, DES, drug-eluting stent(s), FFR, fractional flow reserve, HR, hazard ratio, iFR, instantaneous wave-free ratio, MACE, major adverse cardiac event(s), NHPR, nonhyperemic pressure ratio, PCI, percutaneous coronary intervention, TVF, target vessel failure, VOCE, vessel-related composite event

## Abstract

Coronary physiological assessment using fractional flow reserve or nonhyperemic pressure ratios has become a standard of care for patients with coronary atherosclerotic disease. However, most evidence has focused on the pre-interventional use of physiological assessment to aid revascularization decision-making, whereas post-interventional physiological assessment has not been well established. Although evidence for supporting the role of post-interventional physiological assessment to optimize immediate revascularization results and long-term prognosis has been reported, a more thorough understanding of these data is crucial in incorporating post-interventional physiological assessment into daily practice. Recent scientific efforts have also focused on the potential role of pre-interventional fractional flow reserve or nonhyperemic pressure ratio pullback tracings to characterize patterns of coronary atherosclerotic disease to better predict post-interventional physiological outcomes, and thereby identify the appropriate revascularization target. Pre-interventional pullback tracings with dedicated post-processing methods can provide characterization of focal versus diffuse disease or major gradient versus minor gradient stenosis, which would result in different post-interventional physiological results. This review provides a comprehensive look at the current evidence regarding the evolving role of physiological assessment as a functional optimization tool for the entire process of revascularization, and not merely as a pre-interventional tool for revascularization decision-making.

## Highlights

• Post-PCI physiological assessment provides information about the functional results of revascularization and prognosis after PCI.
• Relative contribution of disease burden in stented and nonstented segments determines the post-PCI physiological indexes and physiological gain from PCI.
• Practical pitfalls of post-PCI FFR or NHPRs should be considered.
• Pre-PCI pullback analysis to characterize the coronary atherosclerotic disease patterns enables better identification of the revascularization target.

## Central Illustration

**Figure:**
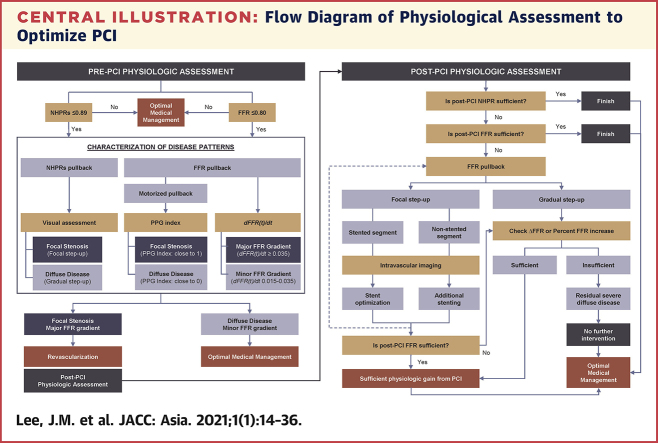

Over the last 2 decades, a wide body of evidence has emerged supporting the use of coronary physiological assessments to define ischemia-causing stenoses and guide coronary interventions. Hyperemic fractional flow reserve (FFR) has been regarded as a reference method to select coronary stenoses that require revascularization, and the recent development of nonhyperemic pressure ratios (NHPRs), such as instantaneous wave-free ratio (iFR), has expanded the applicability of invasive physiological assessment in daily practice (1,2).

However, it should be noted that FFR or NHPR-guided percutaneous coronary intervention (PCI) does not necessarily result in “functionally optimized revascularization,” even after achievement of angiographically successful PCI. In fact, studies have demonstrated that a substantial proportion of patients who underwent angiographically successful PCI had suboptimal post-PCI FFR (3, 4, 5), which has been associated with worse clinical outcomes (3, 4, 5, 6, 7, 8, 9, 10, 11). In this context, efforts to establish the novel role of physiological assessments in functionally optimized revascularization have been undertaken. The concept of “FFR gain” (degree of changes in FFR after PCI) has emerged, and studies have found that both post-PCI FFR value and absolute or relative FFR gain after PCI are significantly associated with improvement of angina severity, quality of life, and future prognosis after revascularization (9,10,12). In addition, recent reports have consistently shown the clinical and prognostic implications of NHPRs in post-PCI physiologic assessment (13, 14, 15, 16).

Achievement of optimal physiological results after PCI should be considered as a fundamental goal of functionally optimized revascularization. Moreover, researchers have tried to characterize disease patterns of coronary atherosclerosis prior to PCI (e.g., focal vs. diffuse disease) using pullback tracings of physiological indexes. Studies have found that the lesions with a major gradient or focal disease determined by pre-PCI pullback tracings have a higher probability of optimal physiological results after PCI than those with a minor gradient or diffuse disease (17, 18, 19). Therefore, this new technique may enable the prediction of expected post-PCI physiological results in the pre-PCI phase, and thereby, improve the selection of more appropriate revascularization targets that would benefit from PCI the most. These recent studies have significantly expanded the potential role of coronary physiological assessments from identification of the ischemia-causing stenoses to optimization of revascularization results, characterization of disease patterns, and selection of more appropriate target lesions for PCI by predicting expected physiological results.

Nevertheless, there has been no standard recommendation or consensus on the proper use of FFR or NHPRs after PCI or the characterization of disease patterns using a pullback analysis. In this regard, proper interpretation of available evidence and a better understanding about the roles of such physiological assessments are crucial in daily practice. Therefore, this review will provide a comprehensive look and analysis of current data including: 1) the evolving role of physiological assessment as a revascularization optimization tool beyond pre-interventional guidance for revascularization decision-making; 2) practical guidance of post-interventional assessment for functionally optimized revascularization; and 3) a novel approach using pullback tracings to optimize selection of the revascularization target.

## Optimal Stent Implantation: Angiography, Intravascular Imaging, and Physiology

Although PCI using current generation drug-eluting stents (DES) has shown efficacy in relieving angina (20,21) and reduced device-related clinical events than bare-metal stents (BMS) or previous generations of DES (22), a substantial proportion of patients continue to experience device-related clinical events or progression of de novo disease (23,24). Despite angiographically successful PCI, nearly one-half of accumulated clinical events during long-term follow-up were derived from previously stented segments (24) and the other half of events were from nonstented segment(s) in the remaining vessels (23).

These 10-year observations imply that angiographically successful PCI has 2 potential limitations: 1) detecting suboptimal procedural results in the stented segment; and 2) detecting residual atherosclerotic or ischemic disease proximal or distal to the stented segment. Studies have shown that about 15% to 20% of patients who underwent angiographically successful PCI showed significant stent underexpansion, malapposition, intra-stent thrombus formation, and edge dissection on intravascular ultrasound (IVUS) or optical coherence tomography (OCT), possibly leading to adverse clinical outcome (25). Although FFR-guided decision of revascularization has significantly reduced the number of stenting and subsequent clinical events than angiography-guided decision-making, continued adverse clinical events after FFR-guided PCI in the FAME (Fractional Flow Reserve Versus Angiography for Multivessel Evaluation) (31.0% at 5 years) (26) and FAME 2 (13.9% at 5 years) (27) trials imply that FFR-guided decision-making followed by angiographic stent implantation does not necessarily translate to functionally optimized PCI. Considering the goal of revascularization is to resolve inducible myocardial ischemia and improve patient prognosis rather than simply alleviate the target coronary stenosis, post-PCI physiological assessment provides crucial information about the effect of revascularization in terms of modifying the degree of vessel-related inducible myocardial ischemia in the subtended myocardium.

## Prognostic Implications of Post-PCI FFR

**Figure 1:**
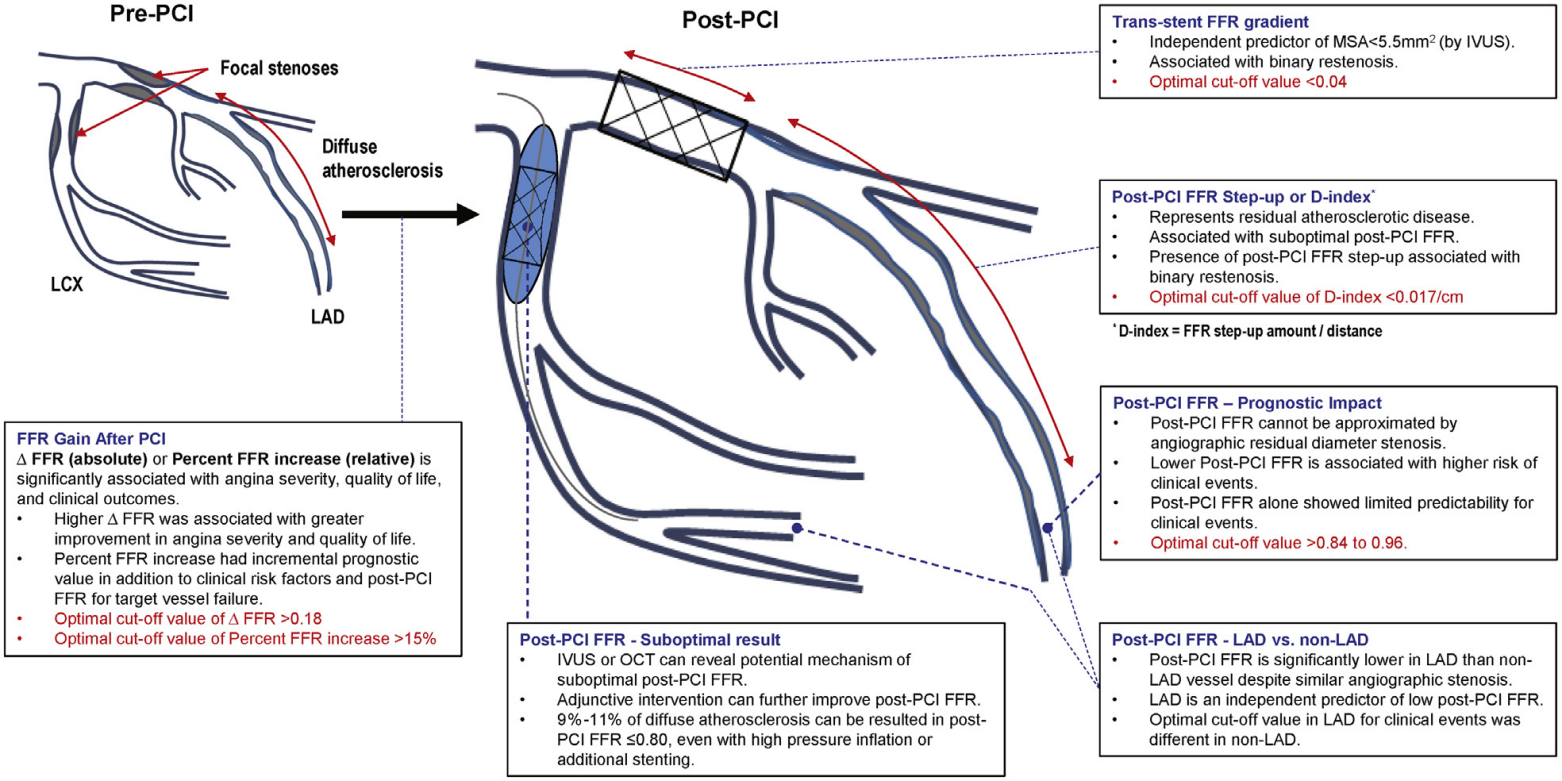
Current Evidence for Clinical Implications of Post-PCI FFR Clinical implications and optimal cut-off values of post-PCI FFR, post-PCI FFR step-up, trans-stent FFR gradient, and FFR gain after PCI are summarized. Each cut-off value indicates a target for optimal post-PCI physiological results and favorable outcomes. FFR = fractional flow reserve; IVUS = intravascular ultrasound; LAD = left anterior descending artery; MSA = minimal stent area; OCT = optical coherence tomography; PCI = percutaneous coronary intervention.

The current recommendations mainly focus on the use of physiological indexes to assess coronary stenoses prior to PCI to aid in the revascularization decision-making process (1,2). However, ample evidence exists showing that post-PCI FFR conveys valuable information on the functional results of the revascularization with prognostic implications (Table 1, Figure 1) (6, 7, 8, 9, 10, 11,28, 29, 30, 31, 32, 33, 34, 35, 36, 37, 38, 39, 40, 41, 42).

**Table 1:** Clinical Studies Evaluating Prognostic Impact of Post-PCI FFR or Nonhyperemic Pressure Ratios

First Author (Ref. #), Year	Inclusion	Device	Follow-Up, Months	Post-PCI Index	Results	Comment
Clinical studies evaluating prognostic impact of Post-PCI FFR						
Bech et al. (28), 1999	60 SIHD with SVD	Balloon angioplasty	24	FFR <0.90	MACE rates between post-PCI FFR <0.90 and ≥0.90 were 41% vs. 12% (p = 0.012).	Post-PCI FFR after successful balloon angioplasty with residual %DS <50% on visual assessment. Post-PCI FFR ≥0.90 was achieved in 48% of patients.
Pijls et al. (29), 2002	750 SIHD or ACS	BMS	6	FFR<0.90	MACE rates were 4.9% in post-PCI FFR >0.95, 6.2% in FFR 0.90–0.95, 20.3% in FFR <0.90, and 29.5% in FFR <0.80.	Post-PCI FFR after successful stent implantation with residual %DS <10% on visual assessment. Post-PCI FFR>0.95 was achieved in 36% and >0.90 in 68% of patients.
Dupouy et al. (30), 2005	100 SIHD with SVD	BMS	6	FFR≤0.95	Post-PCI FFR >0.95 at 6 months showed lower angina frequency than ≤0.95 (19% vs. 52%).	To achieve post-PCI FFR >0.95, higher inflation pressure was needed. Only 52% patients with residual %DS <20% had FFR >0.95.
Klauss et al. (31), 2005	119 SIHD	BMS	6	FFR<0.95	Post-PCI FFR <0.95 was independent predictor of MACE (OR: 6.22; 95% CI: 1.79–1.62; p = 0.004).	Post-PCI FFR after residual %DS <10% on visual assessment. Post-PCI FFR ≥0.95 was achieved in 55% of patients.
Jensen et al. (32), 2007	98 SIHD with SVD	BMS	9	NR	Patients with step-up in FFR between distal vessel and distal stent edge showed higher binary restenosis (44.0% vs. 8.1%; p < 0.001).	Post-PCI FFR was measured after residual %DS 0% on visual assessment. The prognostic impact of residual diffuse atherosclerotic disease after PCI was evaluated by FFR step-up amount in the distal segment of vessel.
Nam et al. (35), 2011	80 SIHD or ACS (nonculprit)	First-generation DES	12	FFR ≤0.90	MACE rates between post-PCI FFR ≤0.90 vs. >0.90 were 12.5% vs. 2.5% (p < 0.01). Target vessel location (LAD vs. non-LAD) was independent predictor of post-PCI FFR.	Post-PCI FFR was measured after residual %DS <20% on visual assessment. Post-PCI FFR >0.90 was achieved in 50% of patients.
Leesar et al. (34), 2011	66 SIHD with SVD	BMS (43%) or first-generation DES (57%)	24	FFR <0.96	MACE rates between post-PCI FFR <0.96 and ≥0.96 were 28% vs. 6% (p = 0.02). After stenting, FFR step-up across the stent was measured in patients with post-PCI FFR <0.96.	If there was trans-stent FFR gradient, further adjunctive balloon was performed using noncompliant balloon. The additional procedure increased the proportion of patients with post-PCI FFR ≥0.96 from 48% to 53%.
Ishii et al. (33), 2011	33 SIHD	First-generation DES	8	NR	Patients with 8-month angiographic restenosis had lower post-PCI FFR (0.81 ± 0.12 vs. 0.92 ± 0.06; p = 0.029). Trans-stent FFR gradient was higher in the restenosis group. Distal segment FFR step-up was not associated with restenosis in stented segment.	Adjunctive high-pressure balloon inflation was performed until angiographic residual stenosis <25%. IVUS-guided DES implantation. Prognostic impact of trans-stent FFR gradient was evaluated.
Ito et al. (36) 2014	97 SIHD or ACS (nonculprit)	Second-generation DES	Median 17.8	FFR ≤0.90	MACE rate was significantly higher in patients with post-PCI FFR ≤0.90 vs. >0.90 were 17% vs. 2% (p = 0.02).	Post-PCI FFR was measured after residual %DS <30% on QCA. IVUS-guided DES implantation. Mainly second-generation DES was used (96.9%).
Doh et al. (37), 2015	107 SIHD or ACS (nonculprit)	First- (60.5%) or second- (39.5%) generation DES	36	FFR <0.89	TVF rates between post-PCI FFR <0.89 vs. ≥0.89 were 11% vs. 39% (p = 0.03). Cut-off of IVUS minimum stent area for post-PCI FFR ≥0.89 was 5.4 mm^2^.	Post-PCI FFR was measured after residual %DS <10% on visual assessment. IVUS-guided DES implantation. Post-PCI FFR ≥0.89 was achieved in 83% of patients.
Reith et al. (38), 2015	66 SIHD	DES	20	FFR ≤0.905	MACE rates between post-PCI FFR ≤0.905 and >0.905 were 35.9% vs. 5.3% (p = 0.01). Cut-off of OCT percent area stenosis after PCI for MACE was ≤16.85%.	Post-PCI FFR was measured after residual %DS <10% on visual assessment. OCT-guided DES implantation. Post-PCI FFR >0.905 was achieved in 61% of patients. DES type was not specified.
Agarwal et al. (6) 2016	574 SIHD or ACS	BMS (21%) or DES (79%)	31	FFR ≤0.86	MACE rates between post-PCI FFR ≤0.86 and >0.86 were 23% vs. 17% (p = 0.02). Subsequent procedure based on post-PCI FFR increased final-PCI FFR from 0.78 ± 0.07 to 0.87 ± 0.05 (42% post dilatation, 33% additional stenting, 18% both dilatation and stenting, 9% IVUS or OCT).	Post-PCI FFR >0.86 was achieved in 68% of patients. FFR <0.86 had incremental prognostic value over clinical and angiographic variables for MACE prediction. DES type was not specified. The authors demonstrated that additional interventions targeting a higher post-PCI FFR would improve patient outcome.
Kasula et al. (39), 2016	390 SIHD and 189 ACS (NSTEMI or UA)	BMS (26%) or DES (74%) in ACS patients	29	FFR ≤0.91 for ACS	Patients with final FFR values >0.91 had significantly less adverse events than those with final FFR ≤0.91 after ACS (19% vs. 30%; p = 0.03). Patients with ACS who achieved final FFR of >0.91 had similar outcomes compared with patients with SIHD (19% vs. 16%; p = 0.51).	This study appears to use a similar database of the study by Agarwal et al. (6) 2016, but focused on ACS population. The authors demonstrated prognostic value of post-PCI FFR in patients with ACS. This study measured FFR in presumed culprit vessel of NSTEMI or UA patients. DES type was not specified.
Piroth et al. (8), 2017	639 SIHD or ACS	First- or second-generation DES	24	FFR <0.92	VOCE rates between post-PCI FFR <0.92 and ≥0.92 were 8.7% vs. 4.2% (HR: 2.14; 95% CI: 1.19 to 3.84; p = 0.011). However, positive likelihood ratio of post-PCI FFR for predicting VOCE was limited (<1.4).	Post-hoc analysis from FAME (69.2% of FFR-guided arm) and FAME 2 (64.2% of FFR-guided PCI plus medical treatment arm) trials.
Li et al. (7), 2017	1476 SIHD or unstable angina	Second-generation DES	36	FFR ≤0.88	TVF rates between post-PCI FFR <0.88 and ≥0.88 were 12.3% vs. 6.1% (p = 0.002), driven by differences in cardiac death (1.9% vs. 0.6%; p = 0.018) and target vessel revascularization (11.8% vs. 5.2%; p = 0.001). Post-PCI FFR <0.88 was independent predictor of TVF at 3 yrs.	Post-PCI FFR was measured after residual %DS <10% on visual assessment. Post-PCI FFR >0.88 was achieved in 67.6% of patients. Post-PCI FFR was measured 10 mm distal to the lesion or stent edge, not at the distal segment of the vessel.
Nishi et al. (12), 2018	716 SIHD	First- or second-generation DES	12	Tertile value of Δ FFR	There was significant association between ΔFFR (post-PCI FFR – pre-PCI FFR) and quality of life (EQ-5D index) at 1 month and 1 yr after PCI (p for trend = 0.047 and 0.009 for 1 month and 1 yr analysis, respectively).	Post-hoc analysis of patient-level pooled data of FAME and FAME 2 trials, similar to the studies by Piroth et al.(8) or Fournier et al. (10). The larger FFR gain from PCI (higher ΔFFR), the greater improvement of quality of life after PCI.
Lee et al. (9), 2018	621 SIHD or ACS (nonculprit)	Second-generation DES	24	FFR<0.84	TVF rates between post-PCI FFR <0.84 and ≥0.84 were 9.1% vs. 2.6%; HR: 3.37; 95% CI: 1.41–8.03; p = 0.006. Percent FFR increase also had prognostic impact (≤15% vs. >15%, 9.2% vs. 3.0%; HR: 3.61; 95% CI: 1.54 to 8.46; p = 0.003). Percent FFR increase had incremental prognostic value in addition to clinical risk factors and post-PCI FFR for TVF prediction.	Post-PCI FFR was measured after residual %DS <20% on visual assessment. Post-PCI FFR ≥0.84 was achieved in 66.0% of patients. Percent FFR increase >15% was achieved in 69.2% of patients. Among the post-PCI FFR ≥0.84 group, there were no significant differences in clinical outcomes according to percent FFR increase
Hwang et al. (11), 2019	835 SIHD or ACS (nonculprit)	Second-generation DES	24	FFR ≤0.84	TVF rates were significantly different between FFR ≤0.84 and >0.84 in all vessels (8.3% vs. 3.1%; p < 0.001). LAD (≤0.82) and non-LAD (≤0.88) showed different best cut-off value for predicting TVF.	Post-PCI FFR was measured after residual %DS <20% on visual assessment.
von Bommel et al. (42), 2019	637 SIHD or ACS	Second generation DES	1	FFR ≤0.90	Post-PCI FFR >0.90 was achieved in 50% of lesions. No significant difference in MACE between post-PCI FFR ≤0.90 and >0.90 at 1 month (2.0% vs. 1.5%; p = 0.636).	Post-PCI FFR was measured using microcatheter system (Navvus RXi, ACIST Medical System) at 20 mm distal from distal edge of the stent. Irrespective of post-PCI FFR, no further treatment was allowed.
Azzalini et al. (40), 2019	65 SIHD or unstable angina	Second-generation DES	12	FFR <0.90	MACE rates between post-PCI FFR <0.90 and ≥0.90 were 31.6% vs. 9.1% (p = 0.047). Residual distal lesion was the most common reason of post-PCI FFR <0.90 (42%), followed by residual uncovered proximal (14%) and distal plaques (2%), stent underexpansion (2%), and edge dissections (2%). No plausible reason was identified in 37%.	Post-PCI FFR was measured using a microcatheter system (Navvus RXi, ACIST Medical System). Operators decided further treatment after post-PCI FFR and then final post-PCI FFR was measured. Post-PCI FFR ≥0.90 was achieved in 33.8% of patients.
Hoshino et al. (41), 2019	201 SIHD with de novo LAD lesions	Second-generation DES	Median 24	FFR <0.86	VOCE was significantly higher if post-PCI FFR <0.86 (log-rank p = 0.002) but not MACE (log-rank p = 0.084). D-index (difference of post-PCI FFR between distal vessel and distal stent edge divided by the length between the 2 points) was significantly lower in vessels with clinical events. The optimal D-index cutoff value for VOCE was 0.017/cm and was a significant predictor for VOCE.	Only LAD lesions were included. Study included patients who underwent IVUS-guided second-generation DES implantation in LAD. The prognostic impact of residual diffuse atherosclerotic disease after successful IVUS-guided PCI was evaluated by the D-index.
Fournier et al. (10), 2019	639 SIHD	First- or second-generation DES	24	Δ FFR ≤ 0.24	The ΔFFR (post-PCI FFR – pre-PCI FFR) was significantly associated with improvement of angina class and the risk of VOCE at 2 yrs. The risk of VOCE was significantly higher in the lowest tertile (ΔFFR ≤0.18) than the highest tertile (ΔFFR >0.31) (HR: 2.01; 95% CI: 1.03–3.92; p = 0.04). ΔFFR ≤0.24 was the most closely associated value with VOCE.	Post-hoc analysis of patient-level pooled data of FAME and FAME 2 trials, similar to the study by Piroth et al. (8) or Nishi et al. (12). The larger FFR gain from PCI (higher ΔFFR), the higher the symptomatic relief and the lower the event rate.
Yang et al. (46), 2020	135 SIHD or unstable angina with LAD lesions	Second-generation DES	72	Trans-stent FFR gradient ≥0.04	Trans-stent FFR gradient ≥0.04 was independent predictor of IVUS MSA <5.5 mm^2^. Patients with trans-stent FFR gradient ≥0.04 showed significantly higher risk of MACE than those with trans-stent FFR gradient <0.04 (30.4% vs. 6.6%; p = 0.031).	Only LAD lesions were included. Study included patients who underwent IVUS-guided second-generation DES implantation in LAD. Prognostic impact of trans-stent FFR gradient was evaluated.
Clinical studies evaluating prognostic impact of Post-PCI nonhyperemic pressure ratios						
Hakeem et al. (13), 2019	574 SIHD or ACS	Second-generation DES	30	FFR ≤0.86 Resting Pd/Pa ≤0.96	MACE rates between post-PCI FFR ≤0.86 and >0.86 were 23% vs. 17% (p = 0.02). MACE rates between post-PCI resting Pd/Pa≤0.96 and >0.96 were 24% vs. 15% (p = 0.0006). Patients with resting Pd/Pa ≤0.96 and FFR ≤0.86 had the highest event rate (25%).	Post-PCI resting Pd/Pa had incremental prognostic value in addition to clinical/angiographic variables and post-PCI FFR for MACE prediction.
Jeremias et al. (14), 2019	500 SIHD or ACS	Second-generation DES	Post-PCI phase	iFR ≤0.89	24.0% of patients showed post-PCI iFR ≤0.89 after operator-judged angiographically successful PCI. Among these patients, iFR pullback showed 81.6% had untreated focal stenosis (38.4% within stent, 31.5% in proximal, 30.1% in distal segment) and 18.4% had diffuse disease. Post-PCI iFR was measured after operator judged the procedure was completed. Post-PCI iFR pullback recording classified the patterns into focal (iFR gradient ≥0.03 within ≤15mm length) or diffuse disease (iFR gradient ≥0.03 over >15mm length).	No clinical outcomes were reported in the initial report.(14) 1-yr clinical outcome was reported in Transcatheter Therapeutics Meeting 2020 (16). Optimal cut-off value of post-PCI iFR was <0.95 (AUC: 0.74; 95% CI: 0.61–0.88) for cardiac death or spontaneous MI and those with post-PCI iFR <0.95 showed significantly higher risk of cardiac death or spontaneous MI (3.2% vs. 0.0%; log rank p = 0.02) or higher risk of cardiac death, spontaneous MI, or clinically driven TVR (5.7% vs. 1.8%; log rank p = 0.04) than those with post-PCI iFR ≥0.95 (16).
Shin et al. (15), 2020	588 SIHD or ACS (nonculprit)	Second-generation DES	24	FFR ≤0.80 Resting Pd/Pa ≤0.92	After angiographically successful PCI, 18.5% had post-PCI FFR ≤0.80 and 36.9% showed post-PCI Pd/Pa ≤0.92. In post-PCI Pd/Pa>0.92 group, 93.8% of patients showed concordant results with post-PCI FFR>0.80. Conversely, in post-PCI Pd/Pa ≤0.92 group, 60.4% of patients showed post-PCI FFR >0.80. Post-PCI FFR ≤0.80 showed significantly higher risk of TVF (10.3% vs. 2.5%; p < 0.001) than >0.80. Post-PCI resting Pd/Pa ≤0.92 showed significantly higher risk of TVF (6.2% vs. 2.5%; p = 0.029) than >0.92. Post-PCI iFR and dPR showed same results with resting Pd/Pa.	Post-PCI resting Pd/Pa and FFR was measured after residual %DS <20% on visual assessment. Among patients with abnormal post-PCI Pd/Pa ≤0.92, only patients with positive post-PCI FFR ≤0.80 showed significantly higher risk of TVF than did those with post-PCI Pd/Pa >0.92. Only post-PCI FFR showed significantly increased reclassification ability for predicting TVF. Only available evidence for prognostic implication of post-PCI iFR and dPR.

ACS = acute coronary syndrome; AUC = area under curve; BMS = bare-metal stent; CI = confidence interval; DES = drug-eluting stent; DS = diameter stenosis; FAME = Fractional Flow Reserve Versus Angiography for Multivessel Evaluation; FFR = fractional flow reserve; HR = hazard ratio; iFR = instantaneous wave-free ratio; IVUS = intravascular ultrasound; LAD = left anterior descending artery; MACE = major adverse cardiac events; NSTEMI = non–ST-segment elevation myocardial infarction; OCT = optical coherence tomography; PCI = percutaneous coronary intervention; Pd/Pa = distal coronary pressure/aortic pressure; QCA = quantitative coronary angiography; SIHD = stable ischemic heart disease; SVD = single vessel disease; TVF = target vessel failure; UA = unstable angina; VOCE = vessel-oriented composite events.

Earlier studies evaluated optimal cut-off values of post-PCI FFR to predict major adverse cardiac event (MACE) in patients who underwent PCI using BMS (28, 29, 30, 31, 32). In these studies, patients with post-PCI FFR ≤0.90 to 0.95 showed significantly higher risk of MACE than those with higher post-PCI FFR. Although these studies had limitations due to inclusion of only single-vessel disease (28,30,32), small number of patients (28,30, 31, 32), or limited follow-up duration (29, 30, 31, 32), they provided unique insights into post-PCI physiological assessments, which were re-validated in later studies in the DES era.

The FFR Post Stent registry evaluated the largest number of patients in the BMS era and first demonstrated the continuous association of higher post-PCI FFR with lower risk of subsequent clinical events after PCI (29). The FROST (French Randomized Optimal Stenting Trial) III registry evaluated post-PCI FFR with stepwise increment of inflation pressures during stent implantation until the target FFR >0.95 was achieved (30). Among patients with angiographic residual diameter stenosis <20%, 48% required further dilatation with higher inflation pressures to reach that goal, and those who achieved post-PCI FFR >0.95 were more likely to be angina-free at 6-month follow-up. This result implies that adjunctive procedural optimization after stenting could achieve higher post-PCI FFR. Jensen et al. (32) evaluated the prognostic impact of FFR step-up between distal vessel and distal edge of the stent and first demonstrated the association of diffuse atherosclerosis in the distal vessel with lower post-PCI FFR, higher FFR step-up in the distal segment of vessel, and higher risk of future restenosis (32).

Later studies evaluated the prognostic impact of post-PCI FFR after DES implantation. Although optimal cut-off values of post-PCI FFR to predict MACE or target vessel failure (TVF) were variable, mostly between 0.89 and 0.91 according to study population and design, most studies presented a similar inverse relationship between post-PCI FFR values and the risk of clinical events (35, 36, 37, 38). Similar findings were also demonstrated in acute coronary syndrome patients excluding ST-segment elevation myocardial infarction (39).

In studies in which second-generation DES were mainly used (7, 8, 9,11,41), the optimal cut-off values of post-PCI FFR to predict TVF were slightly lower than earlier reports with BMS or mainly first-generation DES. The DKCRUSH VII (Double Kissing Crush Versus Provisional Stenting Technique for Treatment of Coronary Bifurcation Lesions VII) study evaluated the largest population (n = 1,476) and reported post-PCI FFR ≤0.88 as an optimal cut-off value for TVF at 3 years (7). It should be noted that post-PCI FFR was measured 10 mm distal to the stent edge in this study. Studies using the rapid exchange microcatheter system (Navvus RXi, ACIST Medical System, Minnesota) reported a similar cut-off value of 0.90 and showed feasibility of microcatheter-based post-PCI FFR measurements (40,42). A post hoc analysis of the FAME and FAME 2 trial population (n = 639) showed a significant difference in vessel-related composite events (VOCE) between post-PCI FFR <0.92 versus ≥0.92 groups (8.7% vs. 4.2%; hazard ratio [HR]: 2.14; 95% confidence interval [CI]: 1.19 to 3.84; p = 0.011). Nevertheless, positive likelihood ratio of post-PCI FFR to predict VOCE was limited (<1.4), suggesting limited predictability for future adverse events (8).

Hwang et al. (11) evaluated 835 patients from Influence of FFR on the COE-PERSPECTIVE (Influence of FFR on the Clinical Outcomes After Percutaneous Coronary Intervention) registry and presented post-PCI FFR ≤0.84 as an optimal cut-off value to predict TVF at 2 years. In this study, the optimal cut-off value of post-PCI FFR was lower in the left anterior descending (LAD) artery (≤0.82) than non-LAD (≤0.88), which was consistently observed in multiple studies (7,8,43,44). Because LAD generally supplies a larger subtended myocardium, which is one of the key determinants of hyperemic coronary flow and consequently FFR, the FFR value can be inherently lower in the LAD than in non-LAD for the same degree of stenosis (45).

Leesar et al. (34) provided a higher cut-off value of post-PCI FFR <0.96 for predicting MACE at 2 years. In this study, trans-stent FFR gradient was measured in patients with post-PCI FFR <0.96, and high-pressure adjunctive balloon inflation was performed if a trans-stent FFR gradient was present. The additional procedure increased the proportion of patients with post-PCI FFR ≥0.96 from 48% to 53%. Ishii et al. (33) further evaluated the clinical implications of trans-stent FFR gradient in addition to post-PCI FFR. In 33 patients who underwent IVUS-guided PCI, patients with angiographic binary restenosis at 8 months (diameter stenosis >50%) showed significantly lower post-PCI FFR (0.81 ± 0.12 vs. 0.92 ± 0.06; p = 0.029) and higher trans-stent FFR gradient (0.08 ± 0.05 vs. 0.03 ± 0.03; p = 0.005) than those without binary restenosis. The prognostic impact of trans-stent FFR gradient was recently re-evaluated by Yang et al. (46), which evaluated 135 patients who underwent IVUS-guided PCI. In this study, the best cut-off value of trans-stent FFR gradient was ≥0.04, which was significantly associated with IVUS-derived MSA <5.5 mm^2^ and a higher risk of MACE.

The prognostic impact of FFR step-up in the distal segment of the vessel, which represents the ischemic burden from residual atherosclerotic disease, was re-evaluated by Hoshino et al. (41), in which the authors presented that D-index (difference in post-PCI FFR between distal vessel and distal stent edge, divided by length between the 2 points) ≥0.017/cm was an independent predictor of VOCE during a median follow-up of 24 months (41).

## Prognostication by FFR Gain After PCI

Previous studies, corroborated by meta-analyses, have consistently shown an inverse relationship between post-PCI FFR and risk of future clinical events (3, 4, 5). However, PCI with stent implantation is a focal treatment, whereas post-PCI FFR reflects both residual stenosis in the stented segment as well as residual disease beyond the stented segment in the target vessel. Therefore, a single cut-off value of post-PCI FFR cannot fully discriminate the relative contribution of stented and nonstented disease burden on patient prognosis. In this regard, quantification of FFR gains from PCI has been suggested to evaluate the contribution of focal stenosis treated by PCI in total cumulative disease burden in the target vessel. FFR gains from PCI can be evaluated by absolute increase of FFR (post-PCI FFR − pre-PCI FFR, so-called ΔFFR) or relative increase of FFR (percent FFR increase: [post-PCI FFR − pre-PCI FFR]/pre-PCI FFR × 100) (9,10,12). Because FFR gain from PCI is influenced by baseline stenosis severity and the relative contribution of disease burden in stented and nonstented segments before and after PCI; this can present a different physiological aspect of a target vessel compared with post-PCI FFR.

Three studies evaluated the concept of FFR gains from PCI and their impact on quality of life (12), angina severity (10), and clinical outcomes (9,10). Lee et al. (9) evaluated 621 patients who underwent PCI based on pre-PCI FFR ≤0.80 from the COE-PERSPECTIVE registry. The best cut-off values of percent FFR increase and post-PCI FFR to predict TVF at 2 years were 15% and 0.84, respectively. Patients with low percent FFR increase (≤15%) or low post-PCI FFR (<0.84) showed a significantly higher risk of TVF than those with high percent FFR increase or high post-PCI FFR, respectively. Percent FFR increase was independently associated with TVF risk and adding percent FFR increase significantly increased discriminant and reclassification ability for the occurrence of TVF compared with a model utilizing post-PCI FFR alone. Nishi et al. (12) evaluated 716 stable ischemic heart disease (SIHD) patients from the combined cohort of FAME and FAME 2 trials and presented that improvement in quality of life at both 1 month and 1 year after PCI was significantly associated with higher ΔFFR or percent FFR increase (12). Fournier et al. (10) evaluated 639 subjects from the same cohort and found that ΔFFR was significantly associated with improvement of angina class and the risk of VOCE at 2 years.

**Figure 2:**
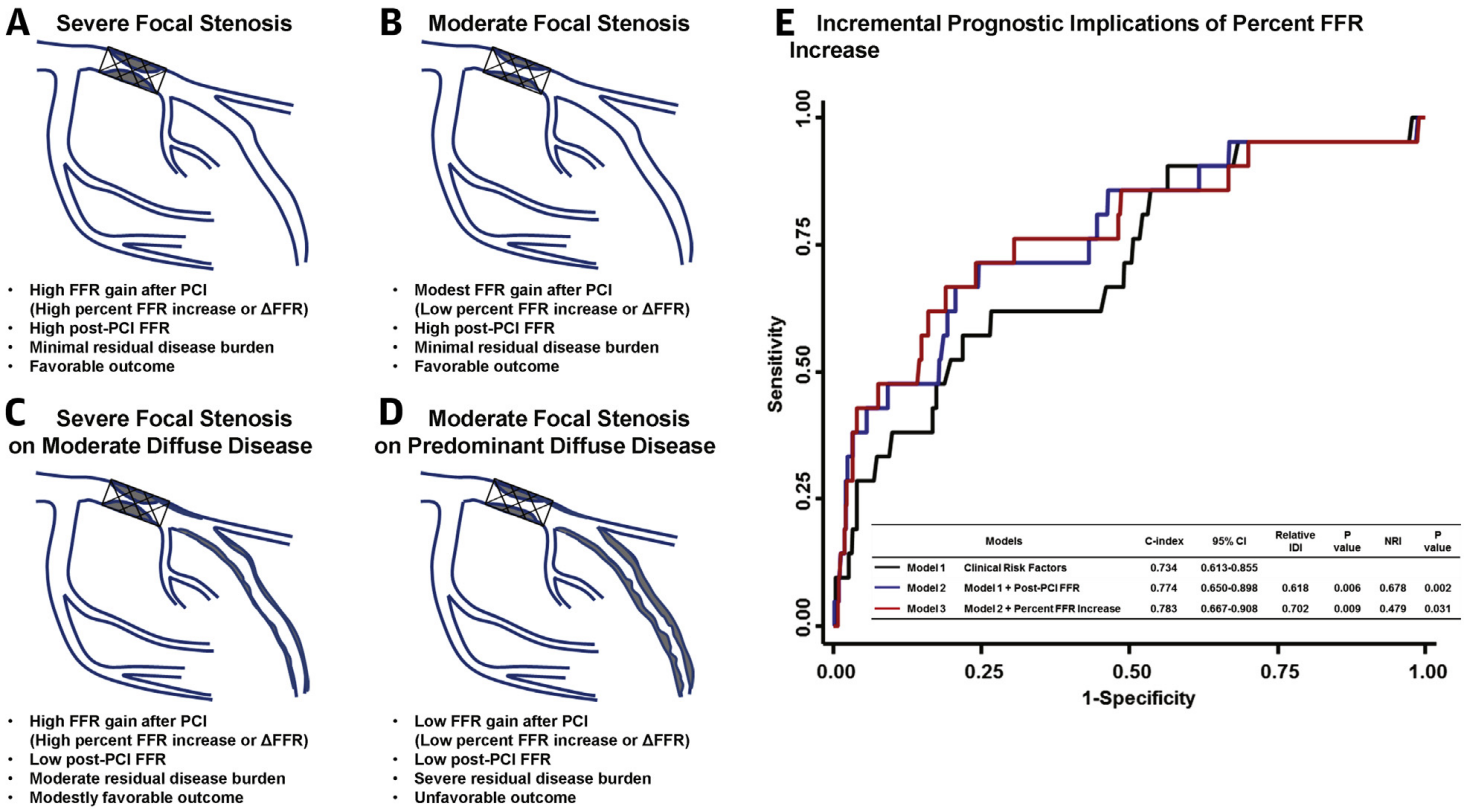
Differential Physiological Response After PCI According to Patterns of Coronary Atherosclerotic Disease **(A to D)** Integrated interpretation with both post-PCI FFR and FFR gain from PCI is important to understand the physiological nature of target vessel disease and discriminate patients who are at higher risk of clinical events. **(E)** The incremental prognostic ability of percent FFR increase in addition to post-PCI FFR was demonstrated in prediction of TVF at 2 years. Adapted with permission from Lee et al. (9). IDI = integrated discrimination improvement; NRI = net classification index; other abbreviations as in Figure 1.

The patterns of FFR gains after PCI (percent FFR increase or ΔFFR) and post-PCI FFR are mainly determined by underlying patterns of coronary atherosclerotic disease or plaque distribution in the target vessels (Figure 2). In patients with mainly focal stenosis (Figures 2A and 2B), percent FFR increase or ΔFFR is determined by pre-PCI lesion severity in case of successful PCI; therefore, there are limited prognostic implications of percent FFR increase or ΔFFR. Conversely, in patients with combined focal stenosis and diffuse atherosclerotic disease (Figures 2C and 2D), FFR gains after PCI and post-PCI FFR are determined by an interaction between the target stenosis and nontarget segments, or by the relative physiological contribution of focal stenosis and diffuse atherosclerotic disease before and after PCI. In patients with higher relative contribution of focal stenosis to vessel-related myocardial ischemia (Figure 2C), PCI will result in a high percent FFR increase or ΔFFR, and post-PCI FFR will be determined by the residual diffuse atherosclerotic disease burden and/or adequacy of stent implantation. Conversely, in patients with higher relative contribution of diffuse atherosclerotic disease than focal stenosis (Figure 2D), PCI will result in low percent FFR increase or ΔFFR and low post-PCI FFR. In this case, the benefit of additional local treatment would be limited, and meticulous secondary prevention and close follow-up are warranted. These results suggest that FFR gains after PCI have independent and incremental prognostic implications to post-PCI FFR (Figure 2E), and an integrated concept of FFR gains after PCI and post-PCI FFR is important in understanding the physiological nature of target vessel disease to better stratify patients at higher risk of clinical events (9).

## Role of Post-PCI FFR to Functionally Optimize Revascularization

**Figure 3:**
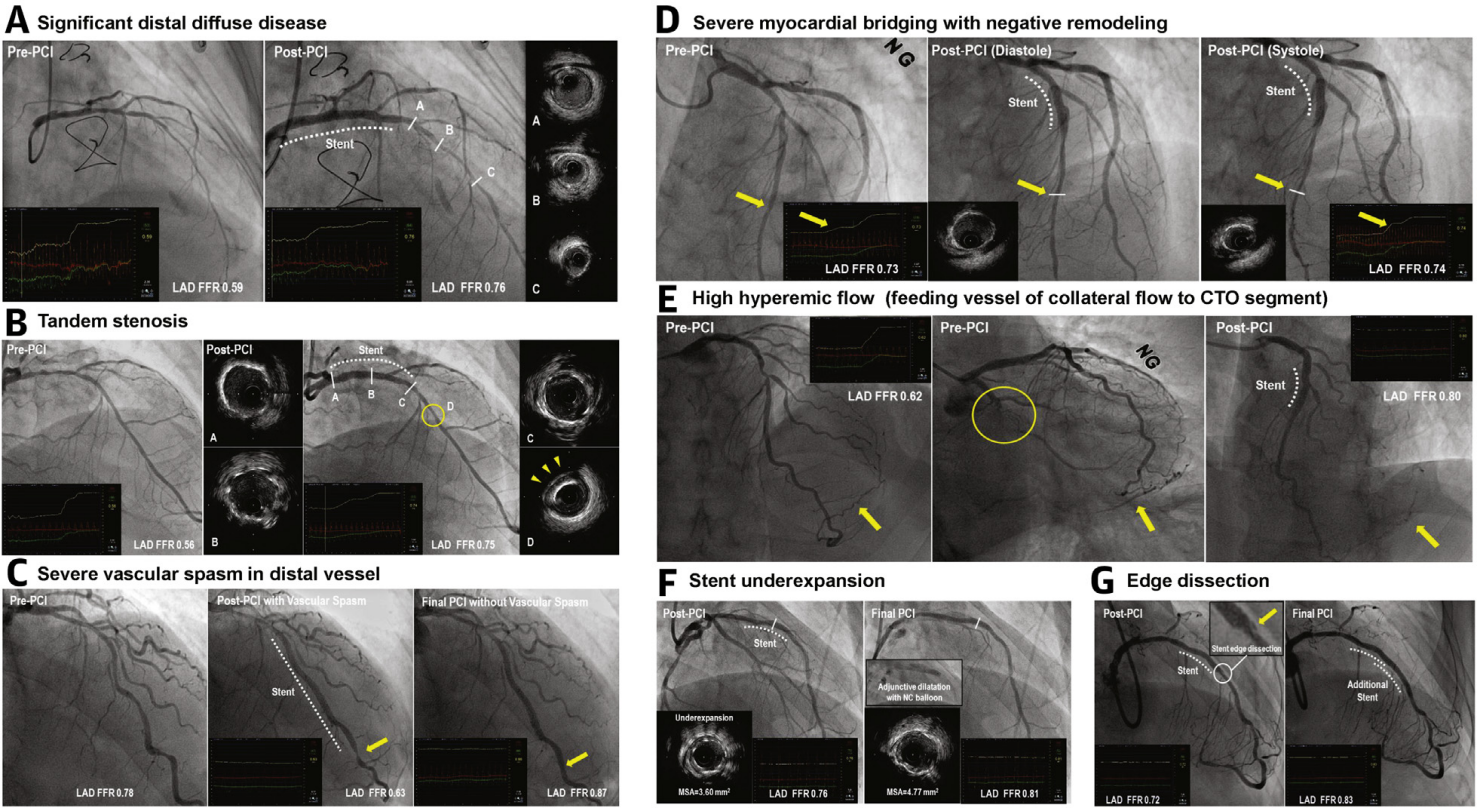
Potential Mechanisms of Suboptimal Post-PCI FFR Illustrative case examples of suboptimal post-PCI FFR are shown. Despite angiographically successful PCI, suboptimal post-PCI FFR can be observed due to **(A)** diffuse atherosclerotic disease in the distal segment of vessel; **(B)** angiographically unapparent tandem stenosis detected by IVUS; **(C)** severe vascular spasm in distal vessel; **(D)** severe myocardial bridging with negative remodeling confirmed by IVUS; or **(E)** increased hyperemic coronary flow in vessel supplying a larger myocardial bed. Suboptimal post-PCI FFR can be also caused by suboptimal stent deployment, such as **(F)** stent underexpansion or **(G)** edge dissection, in which post-PCI FFR was improved after additional intervention. **Arrowsandarrowheads** indicate the potential cause of low post-PCI FFR. Abbreviations as in Figure 1.

Consistent observations of discordance between angiographic and functional results of PCI provoked the idea of using post-PCI FFR as a functional optimization tool, determining whether further intervention was indicated to optimize the final result. Evaluating potential mechanisms for suboptimal post-PCI FFR including pressure drift would be the first step before proceeding with additional intervention(s) (Figure 3).

FFR-SEARCH study reported the potential mechanisms for a suboptimal post-PCI FFR using high-definition IVUS (47). In 100 vessels with a post-PCI FFR ≤0.85, IVUS identified stent underexpansion in 74%; significant residual focal lesion in proximal or distal segment in 29% and 30%, respectively; stent malapposition in 23% (87% of them were accompanied with underexpansion); vascular spasm in 9%; lumen compromising intramural hematoma in 3%; and residual diffuse atherosclerotic disease in 8%. Similarly, Wolfrum et al. (5) reported OCT findings of 21 patients with post-PCI FFR <0.90. Among these, 13 patients (61.9%) showed OCT-defined suboptimal stent deployment and underwent additional interventional procedure. The primary reasons for OCT-defined suboptimal stent deployment were stent underexpansion in 46%, incomplete lesion coverage in 39%, stent malapposition in 54% (all cases of malapposition were accompanied with stent underexpansion and/or incomplete lesion coverage), stent distal edge dissection in 15%, or tissue protrusion in 8%. In 8 patients (38.1%) with post-PCI FFR <0.90, there was no significant issue in the stented segment, but showed diffuse atherosclerotic disease at the distal segment of vessel. After OCT-guided stent optimization, post-PCI FFR was significantly increased from 0.80 ± 0.02 to 0.88 ± 0.01 (p = 0.008).

Regarding stent malapposition, no study has reported its direct association with suboptimal post-PCI FFR. Isolated stent malapposition without other significant suboptimal findings, such as underexpansion, edge dissection, or incomplete lesion coverage, might not be the cause of suboptimal post-PCI FFR, especially in the era of newer-generation DES with thin stent struts (60 to 80 μm). The results from the study by Wolfrum et al. (5) and others also support this hypothesis (6,38). Chung et al. (48) evaluated the impact of stent edge dissection on post-PCI FFR. In 51 patients with angiographically evident stent edge dissection, all type C or D dissections showed post-PCI FFR ≤0.80, and 17.8% of patients with type A or B dissection had post-PCI FFR ≤0.80. Additional stenting was done for stent edge dissection with post-PCI FFR ≤0.80 and mean post-PCI FFR was increased from 0.67 ± 0.12 to 0.88 ± 0.08. The impact of diffuse atherosclerotic disease on suboptimal post-PCI physiological results was demonstrated by multiple studies (32,41,43,44,49). Baranauskas et al. (49) reported that patients with persistently ischemic FFR after stenting showed higher FFR gradient in distal nonstented segment than stented segment (0.11 ± 0.07 vs. 0.08 ± 0.02; p < 0.0001), suggesting that residual diffuse atherosclerotic disease was a main contributor to myocardial ischemia (49). As mentioned in the previous text, independent prognostic implication of D-index also support the importance of evaluating residual diffuse atherosclerotic disease in patients with suboptimal post-PCI physiological results (41). Similarly, a recent study demonstrated that total stent length and post-PCI FFR were the most important predictors for TVF at 2 years (50). These results imply that diffuse atherosclerotic disease confers the risk of both suboptimal post-PCI FFR and subsequent adverse clinical events after PCI.

The concept of post-PCI functional optimization with additional procedures was presented by Agarwal et al. (6) in a larger study cohort than previous studies. Among 574 patients, persistently ischemic post-PCI FFR ≤0.80 was seen in 21% of lesions after angiographically successful PCI. Among these lesions, operators decided to perform adjunctive post-dilatation in 42%, additional stenting in 33%, combined adjunctive post-dilatation and stenting in 18%, or intravascular imaging evaluation in 9%. These additional interventions significantly reduced the proportion of lesions with ischemic FFR from 21% to 9%. In the lesions with persistently ischemic FFR, FFR pullback tracings showed significant FFR step-up at the distal segment of the vessel, suggesting the presence of diffuse atherosclerotic disease in the distal vessel. Recently, a prospective registry of 226 patients evaluated clinical relevance of functional optimization by post-PCI FFR. In this study, 36.5% of revascularized vessels showed immediate post-PCI FFR ≤0.80 (51). Among these vessels, further intervention was decided based on pullback tracing and post-PCI FFR was significantly increased from 0.73 (Q1, Q3: 0.69, 0.77) to 0.80 (Q1, Q3: 0.77, 0.85) after subsequent intervention (p < 0.0001). Another recent randomized controlled trial, TARGET-FFR (An Evaluation of a Physiology-guided PCI Optimisation Strategy) compared the proportion of patients with post-PCI FFR >0.90 between physiology-guided incremental optimization strategy (PIOS) and a blinded post-PCI FFR measurement group. In the PIOS group, additional adjunctive balloon or stenting was performed if focal FFR increase ≥0.05 in the stented or nonstented segment, respectively. Although PIOS failed to achieve the primary endpoint of ≥20% difference in the proportion of patients with post-PCI FFR>0.90 between the 2 groups, the PIOS group showed a significantly lower proportion of patients with post-PCI FFR ≤0.80 than the others (18.6% vs. 29.8%; p = 0.045) (52).

The previously mentioned results clearly support the role of post-PCI FFR as a reliable indicator of suboptimal stent deployment and a signal for the need of a subsequent interventional procedure. When the post-PCI FFR is lower than expected, it is important to evaluate the correctable mechanism for suboptimal post-PCI FFR. In this regard, pressure pullback tracings are helpful to discriminate focal step-up across the stented and/or nonstented segment from diffuse gradual step-up at the distal segment of the vessel. In the presence of focal step-up, intravascular imaging would reveal the target of subsequent interventional procedure. If final post-PCI FFR is still suboptimal despite successful additional procedure, FFR gain from PCI (percent FFR increase or ΔFFR) can provide information about whether physiological gain from stent implantation is sufficiently achieved or possible benefit of additional local treatment still remains (9).

## Prognostic Impact of Residual Anatomic Disease After Functionally-Optimized PCI

Another important question in daily practice is whether residual angiographic stenosis would have prognostic impact after functionally optimized revascularization. Previous studies indicated that residual angiographic disease assessed by residual SYNTAX score (RSS) possessed prognostic impact after angiographically successful PCI (53). However, a post hoc analysis of the FAME trial showed that there was no significant difference in RSS between patients with and without MACE at 1 year after FFR-guided revascularization for multivessel disease (54). In addition, classification by RSS did not show significant difference in MACE risk (log-rank p = 0.55). Similar results were also seen in acute coronary syndrome patients, and the risk of MACE was not different according to RSS after PCI (p = 0.54) (55).

In these studies, FFR-guided revascularization meant that FFR was used to perform (FFR ≤0.80) or defer (FFR >0.80) revascularization. Therefore, it should be noted that FFR-guided revascularization does not necessarily mean that the revascularization was completed with no residual ischemia or functionally optimized results. Considering that about 20% of patients showed post-PCI FFR ≤0.80 even after angiographically successful PCI (6,9), the prognostic impact of residual angiographic disease should be evaluated after functionally optimized revascularization without residual myocardial ischemia defined by post-PCI FFR >0.80. A recent study from the International Post PCI FFR Registry evaluated 1,910 patients with 2,095 revascularized vessels with post-PCI FFR >0.80 (56). TVF at 2 years occurred in 4.9% of the study population. The risk of TVF was not different according to tertiles of RSS (log-rank p = 0.851). Conversely, the risk of TVF was significantly different according to tertiles of post-PCI FFR (log-rank p = 0.009). Furthermore, a multivariable model showed the risk of TVF was significantly associated with post-PCI FFR (HR: 1.091; 95% CI: 1.032 to 1.153; p = 0.002), but not with RSS (HR: 0.969; 95% CI: 0.898 to 1.045; p = 0.417). These differences in the prognostic impact between RSS and post-PCI FFR imply that post-PCI FFR is a better tool to assess residual coronary atherosclerotic disease burden than angiographic assessment and support the importance of functionally optimized revascularization.

## Post-PCI Physiological Assessment Using Nonhyperemic Pressure Ratios

Despite the large body of supporting evidence for physiology-guided PCI and optimization, the penetration rate of FFR in contemporary practice remains limited. A recent nationwide survey well-demonstrated the underlying reasons for not using physiological assessment as being multifactorial, including physician attitude, knowledge barriers, or environmental barriers such as medical cost of pressure wires and hyperemic agents (57). In an effort to simplify the physiological assessment, nonhyperemic coronary pressure–derived parameters have been extensively studied. Given the successful adoption of iFR, the first commercialized NHPRs, various NHPRs have been developed such as resting-full cycle ratio or diastolic pressure ratio (dPR) (58). Later studies consistently presented that these NHPRs, including noncommercialized distal coronary pressure/aortic pressure (resting Pd/Pa) are highly correlated with each other, share identical diagnostic accuracy, and have similar prognostic implications (58,59).

Nevertheless, those studies focused on clinical and prognostic implications of NHPRs in pre-PCI assessment as a tool to guide revascularization decision-making, and there has been limited evidence on the role of NHPRs in post-PCI physiological assessment. Three studies are available to date (Table 1) (13, 14, 15, 16). Hakeem et al. (13) focused on prognostic implications of post-PCI resting Pd/Pa during a median follow-up of 30 months. Among 574 patients who underwent both post-PCI resting Pd/Pa and FFR measurements, the best cut-off values to predict MACE were 0.96 for resting Pd/Pa and 0.86 for FFR. When patients were classified into 4 groups according to post-PCI resting Pd/Pa and FFR, those with suboptimal results in both resting Pd/Pa and FFR showed the highest event rates (25%), conversely, those with resting Pd/Pa >0.96 and FFR >0.86 showed the lowest risk (15%). In comparison of global chi-square values, a predictive model with resting Pd/Pa showed incremental prognostic utility than a model with post-PCI FFR. It should be noted that 45.2% of the patients with suboptimal post-PCI resting Pd/Pa ≤0.96 showed satisfactory post-PCI FFR >0.86; conversely, the proportion of patients with optimal resting Pd/Pa >0.96 but suboptimal FFR ≤0.86 comprised only 7% of the study population. This means that positive predictive value of post-PCI resting Pd/Pa was limited, but negative predictive value of resting Pd/Pa was relatively high to predict post-PCI FFR >0.86.

DEFINE-PCI (Physiologic Assessment of Coronary Stenosis Following PCI) was a single-arm registry that evaluated post-PCI iFR after angiographically successful PCI (14). In this study, 21.9% of vessels (24.0% of patients) showed an ischemic range of post-PCI iFR ≤0.89 despite angiographically successful PCI. Core laboratory analysis of post-PCI iFR pullback recordings classified the disease patterns into focal (iFR gradient ≥0.03 within ≤15 mm length) or diffuse disease (iFR gradient ≥0.03 over >15 mm length). Among patients with post-PCI iFR ≤0.89, 81.6% had angiographically unapparent focal stenosis and 18.4% had diffuse disease. At 1 year from index procedure, the optimal cut-off value of post-PCI iFR was <0.95 (area under the curve 0.74; 95% CI 0.61 to 0.88) for cardiac death or spontaneous MI and those with post-PCI iFR <0.95 showed significantly higher risk of cardiac death or spontaneous MI (3.2% vs. 0.0%; log rank p = 0.02) or higher risk of cardiac death, spontaneous MI, or clinically driven TVR (5.7% vs. 1.8%; log rank p = 0.04) than those with post-PCI iFR ≥0.95 (16).

**Figure 4:**
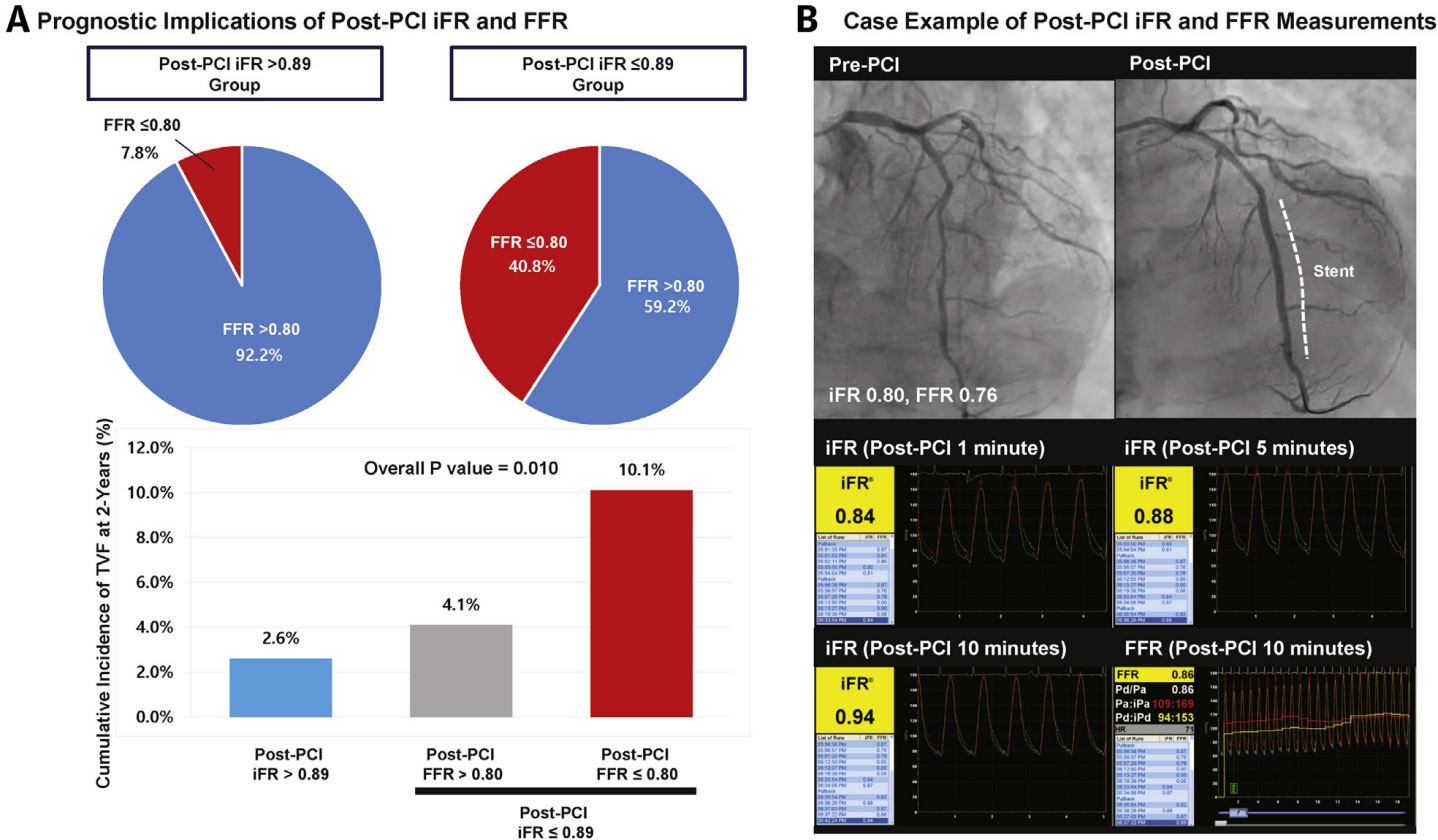
Practical Use of Nonhyperemic Pressure Ratios in Post-PCI Physiological Assessment **(A)** Majority of post-PCI iFR >0.89 group showed post-PCI FFR >0.80; however, 59.2% of patients with post-PCI iFR ≤0.89 had post-PCI FFR >0.80 **(upper panel).** Only patients with both ischemic post-PCI iFR and FFR showed significantly increased risk of 2-year TVF, but not those with post-PCI iFR ≤0.89 and post-PCI FFR >0.80 **(lower panel).** Adapted and modified with permission from Shin et al. (15). **(B)** Illustrative case example presents the influence of post-occlusion reactive hyperemia and gradual increase of post-PCI iFR from 1 to 10 min after PCI. Rechecking by post-PCI FFR confirmed satisfactory post-PCI physiological results. TVF = target vessel failure; other abbreviations as in Figure 1.

In PERSPECTIVE-PCI (Prognostic Perspective of Invasive Hyperemic and Non-Hyperemic Physiologic Indices Measured After Percutaneous Coronary Intervention), a multicenter registry of 588 patients who underwent angiographically successful PCI, there was a significant difference in TVF according to the ischemic cut-off values of post-PCI Pd/Pa and FFR (0.92 and 0.80, respectively). More importantly, 26.3% of the total study population showed discordance between post-PCI resting Pd/Pa and post-PCI FFR. In patients with post-PCI Pd/Pa >0.92, 93.8% showed concordant post-PCI FFR (>0.80); yet, 60.4% of patients with post-PCI Pd/Pa ≤0.92 showed discordant post-PCI FFR (>0.80). Regarding clinical outcomes, only patients with concordantly suboptimal post-PCI Pd/Pa ≤0.92 and FFR ≤0.80 had increased risk of TVF. In contrast, patients with suboptimal post-PCI Pd/Pa ≤0.92 but negative FFR >0.80 had similar risk of TVF compared with those with post-PCI Pd/Pa >0.92. In this study, post-PCI iFR and dPR were calculated using resting Pd/Pa recordings and showed similar results (Figure 4A).

The previously mentioned results imply that post-PCI physiological assessment can be initiated with measuring post-PCI NHPRs. As seen in both Hakeem et al. (13) and PERSPECTIVE-PCI results (15), almost all patients with nonischemic range of NHPRs (post-PCI resting Pd/Pa >0.92 or iFR >0.89) had post-PCI FFR >0.80. Considering that post-PCI NHPRs measurement does not require administration of hyperemic agents, saves additional procedure time, and reduces patient discomfort, post-PCI NHPRs may serve as a gatekeeper for additional measurement of post-PCI FFR with induction of hyperemia.

## Practical Insights for Post-PCI Physiological Assessment With FFR or NHPRs

Although the previously mentioned studies support the prognostic implications of post-PCI FFR or NHPRs, some practical points should be reviewed. Any invasive physiological index can be influenced by transient changes in boundary conditions such as driving pressure, microcirculatory resistance, or coronary flow. Because microcirculatory resistance is the main determinant of coronary flow through an epicardial artery, procedure-related transient microcirculatory dysfunction can influence post-PCI FFR value. Multiple studies indicated transient changes in hyperemic microcirculatory resistance after PCI and its influence on myocardial perfusion imaging (60) and post-PCI FFR (61). Murai et al. (61) compared post-PCI FFR values according to index of microcirculatory resistance (IMR) measured after successful elective stenting. There was a significant linear association between post-PCI IMR and FFR values, and IMR was an independent predictor of post-PCI FFR. These results imply that prognostic stratification by post-PCI FFR or FFR gain from PCI can be limited in the presence of combined severe microvascular disease or transient microvascular dysfunction after PCI. Therefore, caution is needed to interpret post-PCI FFR if significant microvascular dysfunction, such as no-reflow or slow-flow, occurred during the procedure.

Post-PCI NHPRs can be also influenced by transient changes in boundary conditions such as increased heart rate, elevated sympathetic tone, or post-occlusion reactive hyperemia. In PERSPECTIVE-PCI registry, patients with post-PCI resting Pd/Pa ≤0.92 but FFR >0.80 showed significantly shorter resting mean transit time (Tmn), but similar hyperemic Tmn compared with those with post-PCI resting Pd/Pa >0.92 (15). These results suggest that abnormal post-PCI resting Pd/Pa values might not be caused entirely by residual ischemia but also by increased resting coronary flow in the post-PCI phase. Previous studies consistently demonstrated the presence of post-occlusion reactive hyperemia (62,63), and it occurred even after brief coronary occlusions <2 s in duration (62). Therefore, any post-PCI NHPRs can be influenced by the presence of post-occlusion reactive hyperemia, which can result in discordance between post-PCI NHPRs and FFR (Figure 4B). Because there is no available method to confirm the recovery of resting status after PCI, re-checking post-PCI FFR would be warranted in patients with no reasonable explanation from angiographic or intravascular imaging findings for ischemic range of post-PCI NHPRs. If NHPRs are the only available option, the operator should be more cautious to ensure sufficient recovery time before measuring the NHPRs.

## Prediction of Post-PCI Physiological Results Based on Pre-PCI Assessment

Another important advance in physiologic assessment is prediction of expected post-PCI values based on pre-PCI pullback method (Table 2). The efforts to predict post-PCI results have been started to simplify the procedure in serial or tandem stenoses (64).

**Table 2:** Clinical Studies Evaluating Clinical Role of Pullback Tracing

First Author (Ref. #), Year	Inclusion	Physiological Index	Study Methods	Results	Comment and Implications
Pijls et al. (65), 2000	32 patients with 2 tandem stenoses in 1 vessel	FFR Manual pullback	Apparent FFR was calculated using proximal and distal pressure across the stenosis without consideration of collateral flow. Predicted FFR was calculated under consideration of collateral flow (coronary wedge pressure). True FFR of each stenosis was directly measured after PCI of other stenosis.	True FFR (0.78 ± 0.11) and predicted FFR with consideration of collateral flow (0.78 ± 0.12) were similar (r = 0.92). However, apparent FFR without consideration of collateral flow (0.85 ± 0.08) was significantly overestimated (p < 0.01).	This study documented that: 1) there is hemodynamic interaction of tandem stenoses in hyperemic condition; and 2) to assess the true physiological severity of each stenosis in tandem stenoses, the influence of collateral flow by measuring coronary wedge pressure during PCI of 1 stenosis was required.
Kim et al. (66), 2012	131 patients with multiple intermediate stenoses in 1 vessel	FFR Manual pullback	Apparent FFR was calculated using proximal and distal pressure across the stenosis without consideration of collateral flow. Stenosis with larger FFR step-up amount was treated first. True FFR of secondary stenosis was directly measured after PCI of primary stenosis.	Apparent FFR underestimates true lesion severity (apparent FFR was higher than true FFR after PCI of primary stenosis). After treating first stenosis with larger FFR step-up amount among vessels with initial FFR <0.80, 80.9% of vessels were deferred based on post-PCI FFR ≥0.80. Only 1 TVR occurred due to in-stent restenosis. There was no event related with deferred lesion during median follow-up of 501 days.	This study demonstrated that: 1) treatment strategy of tandem stenosis can be guided by FFR pullback tracing and amount of FFR step-up across the stenosis; 2) repeated measurement of FFR after PCI of the primary target stenosis is required to accurately assess the functional significance of secondary stenosis due to hemodynamic interaction among the stenoses in hyperemic condition; and 3) after treating stenosis with larger FFR step-up, remaining lesion with FFR >0.80 can be safely deferred.
Park et al. (67), 2012	52 patients with tandem stenoses for which FFR in vessel ≤0.80	FFR Manual pullback	Stenosis with larger FFR step-up amount was treated first, and then FFR was remeasured in the vessel. Second stenosis was treated if post-PCI FFR ≤0.80 after treatment of first stenosis.	After treating stenosis with larger FFR step-up amount, 27% of remaining lesions showed FFR >0.80 and deferred. During 9-month follow-up, only 1 TVR occurred.	After treating stenosis with larger FFR step-up, remaining lesion with FFR >0.80 can be safely deferred.
Nijjer et al. (17), 2014	32 vessels with tandem or diffuse disease	iFR Motorized pullback (0.5 mm/s)	Based on iFR step-up amount, post-PCI predicted iFR was calculated and compared with observed post-PCI iFR values.	Predicted post-PCI iFR 0.94 ± 0.01 vs. observed post-PCI iFR 0.93 ± 0.01. Correlation coefficient between predicted iFR step-up amount and observed iFR step-up amount was 0.97 (p < 0.001).	In resting condition, changes in coronary flow and trans-stenotic pressure gradient after PCI are relatively stable compared with hyperemic condition (17,70). This study demonstrated that functional significance of each stenosis in tandem stenoses can be intuitively approximated by adding iFR step-up amount in pullback recording into pre-PCI iFR value without the need for measuring balloon occlusion pressure or using complex equations.
Kikuta et al. (70), 2018	128 patients with tandem or diffuse disease	iFR Manual pullback (96.4%) or Motorized pullback 0.5–1.0 mm/s (3.6%)	1. Based on iFR step-up amount, post-PCI predicted iFR was calculated and compared with observed post-PCI iFR values. 2. Number of significant lesions and total lesion length to treat presumed by operator was compared before and after iFR pullback information	1. Predicted post-PCI iFR 0.93 ± 0.05 vs. observed post-PCI iFR 0.92 ± 0.06. Correlation coefficient between 2 values was 0.73 (p < 0.001) 2. After iFR pullback information was given, 31% and 72% of operator’s judgment was changed regarding number of significant lesions and total lesion length to treat, respectively.	This study re-evaluated previous results from hypothesis-generating study (17) from multicenter prospective registry. Although absolute difference between predicted post-PCI iFR and observed post-PCI iFR was small, the correlation between the 2 values was modest. This study also demonstrated iFR pullback can change operator’s judgment about number of significant lesions and total lesion length to treat.
Kawase et al. (71), 2018	71 patients	iFR Manual pullback	Based on iFR step-up amount, post-PCI predicted iFR was calculated and compared with observed post-PCI iFR values.	1. Predicted post-PCI iFR 0.95 ± 0.05 vs. observed post-PCI iFR 0.91 ± 0.06. Correlation coefficient between 2 values was 0.76 (p < 0.001) 2. iFR gradient across the implanted stent was only independent predictor of failed post-PCI iFR prediction (defined as >0.036 difference between predicted and observed iFR values).	This study demonstrated the significant difference between predicted post-PCI iFR and observed post-PCI iFR in real world practice. As with Kikuta et al. (70), the correlation between the predicted and observed value was modest. Residual iFR gradient across the implanted stent could be major cause of difference between predicted and observed values of post-PCI iFR.
Collet et al. (18), 2019	117 patients	FFR Motorized pullback (1mm/sec)	In motorized FFR pullback curve, the authors acquired 2 quantitative values and calculated summary index. 1. Maximum value of FFR gradient over 20 mm 2. Length of functional disease, which defined the distance between the points with FFR drop ≥0.0015/mm 3. Using those 2 acquired values, the authors defined PPG (hyperemic pullback pressure gradient) index as a ratio between maximum FFR gradient over 20 mm and FFR gradient in total vessel (1 – distal FFR), modified by relative length of functional disease to total vessel length.	1. The authors compared classification of disease pattern (focal, diffuse, or mixed) between angiographic assessment and FFR pullback assessment. Decision about focal, diffuse, and mixed was made subjectively by 2 independent researchers. 36% of the cases showed discordant classification. 2. Same comparison was done between angiographic assessment and PPG index-based classification. 53% of cases showed discordant classification. 3. PPG index-based classification of disease pattern was made based on tertile values of PPG index.	This study demonstrated that disease pattern assessed by motorized FFR pullback could be different with angiographic assessment only. Prognostic impact of PPG index-based focal, diffuse, and mixed disease patterns needs to be validated. Motorized FFR pullback maneuver in daily practice can be challenging and has several technical limitations, such as prolonged adenosine infusion and need for additional pullback devices.
Cook et al. (72), 2019	640 patients	iFR Manual pullback	691 iFR pullback tracings from different vessels were independently analyzed by: 1) 15 human expert; 2) heart team; and 3) algorithmic interpretation regarding hemodynamic appropriateness of PCI and extent of PCI based on iFR pullback tracings.	Algorithmic interpretation showed noninferior performance in determining hemodynamic appropriateness of PCI with human expert or heart team. Of note, algorithmic interpretation detected 31.4% of 691 traces had significant pressure drift. Heart team misclassified hemodynamic appropriateness of PCI in 15% of cases due to missed significant pressure drift, but there was no misclassified case by algorithmic interpretation.	This study showed algorithmic interpretation of iFR pullback curve resulted in: 1) noninferior judgment about hemodynamic appropriateness of PCI and PCI strategy than human experts; and 2) more reproducible and objective interpretation of iFR pullback tracings. Further study is needed to clarify clinical and prognostic implications of algorithmic interpretation of iFR pullback tracings.
Warisawa et al. (76), 2020	70 patients with tandem stenoses in 1 vessel	iFR and FFR Motorized pullback (0.5–1.0 mm/s)	88 vessels with tandem stenoses were evaluated by both iFR and FFR pullback. In tandem stenoses, predominant lesion was determined by higher step-up of physiologic indices (iFR and FFR) across the stenosis.	In comparison of classification for predominant stenosis, iFR and FFR pullback showed 22.7% discordance.	This study demonstrated that iFR and FFR pullback can disagree for defining predominant stenosis in about 20% of tandem stenoses. Further study is warranted to clarify prognostic impact of the disagreement and which method would be more clinically useful.
Lee et al. (19), 2020	516 patients	FFR Manual pullback (5–6 mm/s)	Using pre-PCI FFR pullback tracings, instantaneous FFR gradient per unit time (*dFFR(t)/dt*). Cut-off values of *dFFR(t)/dt* to define major and minor FFR gradient were ≥0.035/s and ≥0.015/s, respectively. According to the presence of major or minor FFR gradient lesion, disease patterns were classified into major, minor, and mixed FFR gradient group. Post-PCI physiologic results (post-PCI FFR and percent FFR increase) was compared according to *dFFR(t)/dt*-based classifications (major, minor, and mixed FFR gradient group). *dFFR(t)/dt* value was compared between motorized pullback (1 mm/s) and manual pullback, which showed high correlation (r = 0.980) without significant difference (absolute difference 0.001; p = 0.828).	In validation cohort from single center with standardized pullback method with constant speed (5–6 mm/s), major FFR gradient group showed significantly higher post-PCI FFR and percent FFR increase and minor FFR gradient group showed the lowest post-PCI FFR and percent FFR increase. The results were the same in the external validation cohort with heterogenous manual pullback methods. The proportions of suboptimal post-PCI physiological results were significantly different among the 3 groups (10.4% vs. 25.8% vs. 45.7% for the major, mixed, and minor FFR gradient groups, respectively; p < 0.001) in validation cohorts. Absence of major FFR gradient lesion (OR: 2.435; 95% CI: 1.252 to 4.734; p = 0.009) was an independent predictor for suboptimal post-PCI physiological results.	This study demonstrated that manual FFR pullback-derived *dFFR(t)/dt* could discriminate disease patterns into major, minor, and mixed FFR gradient group and post-PCI physiologic results (post-PCI FFR and percent FFR increase) can be predicted according to *dFFR(t)/dt*-based classifications. Furthermore, the results using *dFFR(t)/dt* were not different according to motorized pullback (1 mm/s), constant manual pullback from single center, and heterogenous pullback from multicenter registry data, which support clinical usability and generalizability of this method. Further study is warranted to evaluate the prognostic impact of *dFFR(t)/dt*-based treatment strategy.

OR = odds ratio; PPG = hyperemic pullback pressure gradient; TVR = target vessel revascularization.

**Figure 5:**
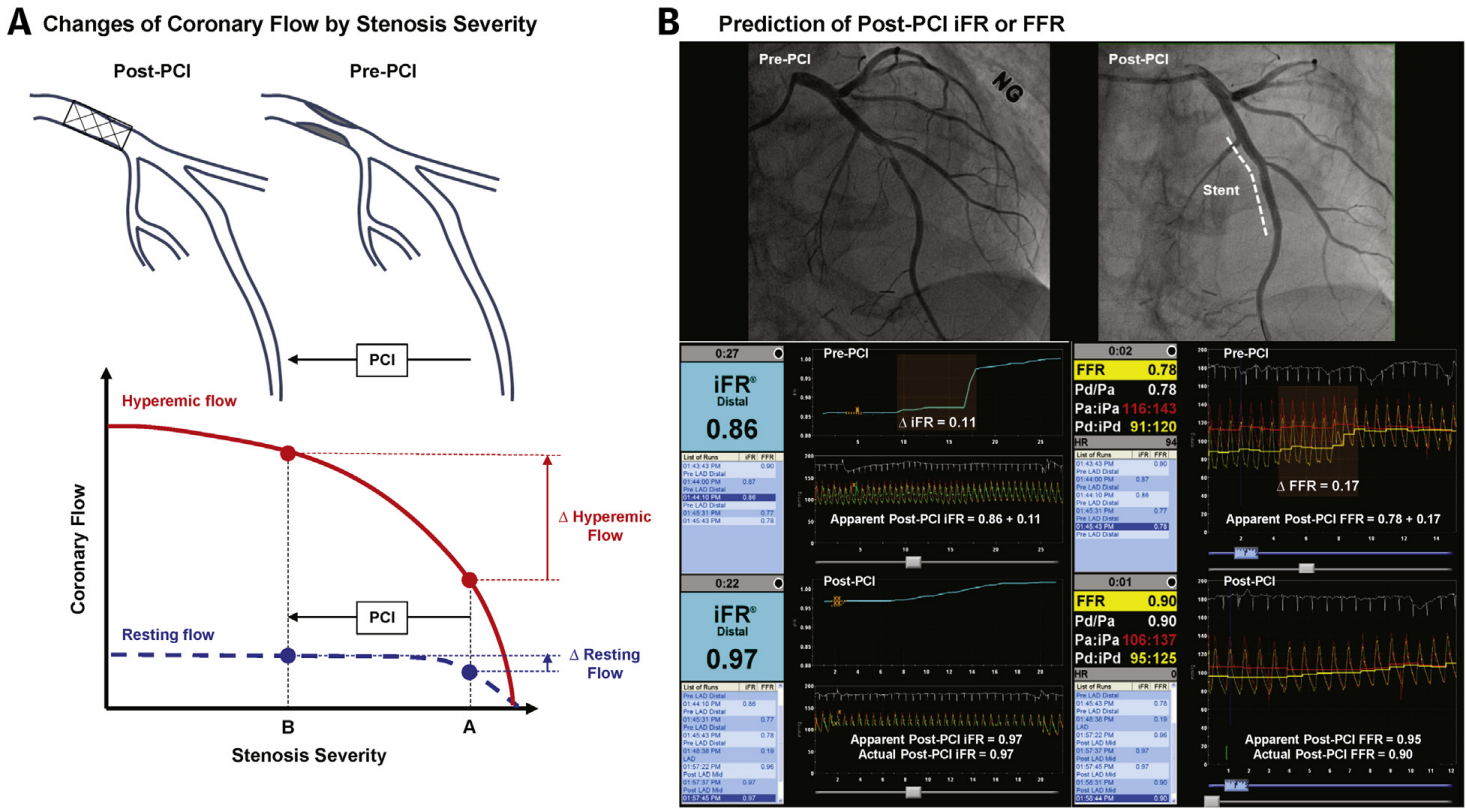
Prediction of Post-PCI Physiological Results Based on Pre-PCI Assessment **(A)** In hyperemic status, coronary flow sensitively changes according to stenosis severity, and removing stenosis by PCI will increase hyperemic flow and trans-stenotic pressure gradient across the secondary stenosis **(red solid line)**. Conversely, coronary flow relatively maintains stable in resting status until critical stenosis develops; therefore, PCI induces relatively smaller changes in coronary flow with predictable change in trans-stenotic pressure gradient **(blue dotted line)**. **(B)** Post-PCI iFR value could be predicted by adding step-up amount in pullback curve into pre-PCI iFR value (apparent value 0.86 + 0.11 = 0.97), which was similar to the actual post-PCI iFR value (0.97) **(left panel)**. However, apparent FFR value (0.78 + 0.17 = 0.95) underestimated the actual post-PCI FFR value (0.90) **(right panel).** Abbreviations as in Figure 1.

During maximal hyperemia, coronary flow is affected by changes in stenosis severity, and removing a stenosis by PCI will increase hyperemic flow and the trans-stenotic pressure gradient across the remaining stenosis (Figure 5A) (64, 65, 66). Even with a single angiographic stenosis, underlying diffuse atherosclerotic disease, which is often angiographically unapparent, can cause a cumulative effect in inducible flow limitation across the vessel. Therefore, true functional significance of secondary stenosis or underlying diffuse atherosclerotic disease can be underestimated in the presence of primary stenosis. This effect was well demonstrated by an initial report by Pijls et al. (65), in which the pressure gradient across the secondary stenosis was significantly increased after relieving primary stenosis (from 10 ± 7 mm Hg to 19 ± 11 mm Hg). A later report by Kim et al. (66) showed similar results of increased pressure gradient across the secondary stenosis after stenting for primary stenosis (from 7.7 ± 5.9 mm Hg to 10.9 ± 7.8 mm Hg) (66). This means that actual post-PCI FFR would be lower than apparent post-PCI FFR (pre-PCI FFR+FFR step-up amount across the primary stenosis) (Figure 5B). Although additional accounting of balloon occlusion pressure of primary stenosis (coronary wedge pressure) can provide a more accurate prediction of FFR for the secondary stenosis, a practical application would be difficult, and the equation cannot be applied in the presence of a large side branch between the stenoses (65,66). In this regard, repeated measurements of FFR after stenting the primary stenosis has been a standard recommendation to accurately assess the functional significance of residual disease (66,67).

Conversely, coronary flow maintains relatively stable in nonhyperemic status according to stenosis severity until critical stenosis develops (68,69). As illustrated in Figure 5A, a relatively smaller change in coronary flow under nonhyperemic conditions induces more predictable change in trans-stenotic pressure gradient and NHPRs after PCI (69). This means that post-PCI value of NHPRs can be intuitively approximated by adding a step-up amount in pullback recording into pre-PCI value without the need for measuring balloon occlusion pressure or complex equation (Figure 5B). Therefore, procedural planning based on predicted post-PCI NHPRs value using pre-PCI pullback may be possible. With this theoretical background, multiple studies compared apparent post-PCI iFR (pre-PCI iFR + iFR step-up amount across the stenosis) and actual post-PCI iFR (17,70,71).

In a pilot study by Nijjer et al. (17), iFR pullback was done in 32 vessels using a motorized pullback device (0.5 mm/s). The predicted and observed iFR step-up amount showed close correlation (r = 0.97; p < 0.001) and the difference between predicted and observed iFR was 0.016 ± 0.004. In the iFR GRADIENT (Single instantaneous wave-Free Ratio Pullback Pre-Angioplasty Predicts Hemodynamic Outcome Without Wedge Pressure in Human Coronary Artery Disease) study, predicted and observed post-PCI iFR values were similar (0.93 ± 0.05 vs. 0.92 ± 0.06) but with a slightly lower correlation (r = 0.73; p < 0.001) (70). It should be noted that 96.4% of patients in this study were evaluated by manual pullback. Kawase et al. (71) further evaluated the main reason for the difference between predicted and observed post-PCI iFR values. In this study, iFR gradient across the implanted stent was the only independent predictor of failed post-PCI iFR prediction, which was defined as >0.036 difference between the 2 values (71). This result implies that predicted iFR value could be regarded as the best expected value of post-PCI iFR, assuming stent implantation will result in no iFR gradient across the stented segment. Given its simplicity and intuitive nature, this approach can be adopted in daily practice to provide an optimal target value of post-PCI iFR. In addition, a coregistration system incorporating both iFR pullback and angiographic images (SyncVision, Philips/Volcano, Amsterdam, the Netherlands) enables convenient procedural planning based on predicted iFR values. Nevertheless, further study is needed to clarify prognostic implications of NHPR-based procedural planning and revascularization. The currently ongoing DEFINE-GPS study will clarify the prognostic impact of iFR pullback and iFR-angiography coregistration system for TVF at 2 years compared with angiography guidance in determining PCI strategy (NCT04451044).

## Characterizing Coronary Atherosclerotic Disease to Optimize Revascularization

Another important question in daily practice would be how to better identify lesions with expected suboptimal post-PCI physiological results, where local treatment with stent implantation would not provide significant benefits. In this regard, there are continuous efforts to characterize patterns of coronary atherosclerotic disease and discriminate physiological “focal” stenosis from “diffuse” lesions. Although currently available studies used different physiological indexes and methodologies, the main purpose of these studies was to find the optimal candidate for revascularization who would be expected to have optimal post-PCI physiological results and better prognosis after PCI.

**Figure 6:**
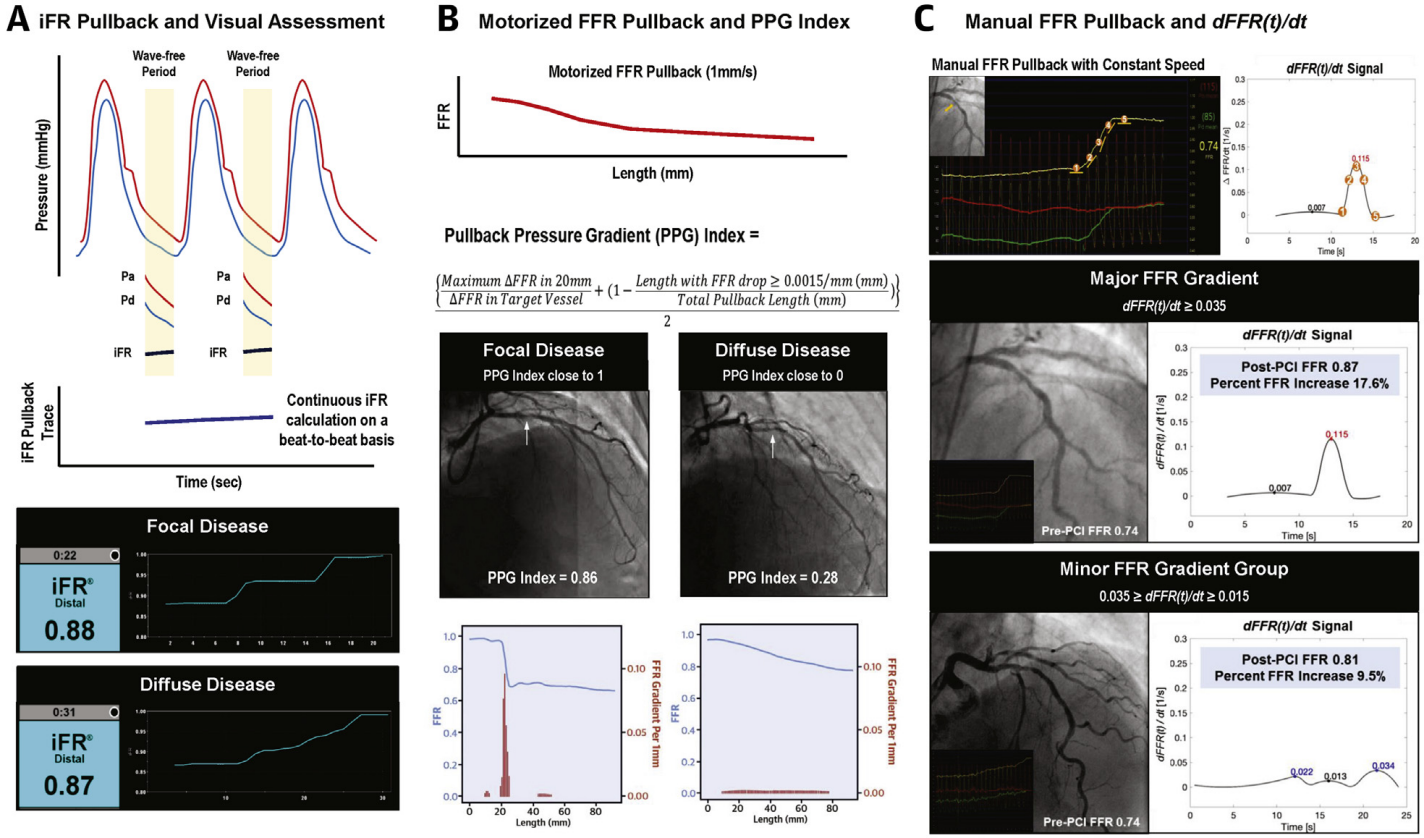
Physiological Index Pullback to Characterize Patterns of Coronary Atherosclerotic Disease **(A)** iFR pullback curve displays continuous iFR values on a beat-to-beat basis along the length of vessel. By visually inspecting the step-up patterns, it is possible to discriminate focal versus diffuse disease. **(B)** Motorized FFR pullback recordings enable the calculation of PPG index. This method quantifies the coronary atherosclerotic disease pattern and discriminates focal (PPG index close to 1) from diffuse disease (PPG index close to 0). **White arrow** indicates the target lesion in the angiography. Adapted with permission from Collet et al. (18). **(C)** The instantaneous FFR gradient per unit time, *dFFR(t)/dt*, quantifies the amount of FFR changes across the target stenosis. Using cut-off values of ≥0.035/s for major FFR gradient and ≥0.015/s for minor FFR gradient, coronary atherosclerotic disease patterns and probability of optimal post-PCI physiological results can be differentiated. Adapted with permission from Lee et al. (19). *dFFR(t)/dt =* instantaneous fractional flow reserve gradient per unit time; PPG = pullback pressure gradient; other abbreviations as in Figure 1.

Figure 6 depicts 3 different methods to characterize patterns of coronary atherosclerotic disease. The iFR pullback displays continuous values on a beat-to-beat basis along the length of vessel and is currently commercially available (Figure 6A) (17). Although the DEFINE PCI study proposed quantitative criteria to discriminate focal (iFR gradient ≥0.03 within ≤15 mm length) or diffuse disease (iFR gradient ≥0.03 over >15 mm length) in post-PCI iFR pullback recording (14), the judgment in pre-PCI phase was mainly performed by visual assessment of iFR pullback curves (72, 73, 74). Nevertheless, the iFR GRADIENT study well demonstrated that the information from pre-PCI iFR pullback significantly changed treatment plan by modifying the number of significant lesions and total lesion length to treat in 31% of interrogated vessels (70). However, the subjective nature and interobserver variability in assessment of disease pattern based on iFR pullback curve should be noted. Warisawa et al. (74) presented more than 20% of assessments by non-experts misinterpreted the disease pattern when judged by expert consensus (74). To overcome variability in assessing the iFR pullback curve, Cook et al. (72) evaluated the performance of artificial intelligence–based interpretation. In their study, artificial intelligence interpretation was noninferior and more reproducible compared with that of expert interventional cardiologists in determining both the hemodynamic appropriateness for PCI and the optimal physiological PCI strategy.

The second method is motorized FFR pullback and quantification of pullback pressure gradient index (Figure 6B). Collet et al. (18) used motorized FFR pullback with 1 mm/s and acquired 2 quantitative parameters: 1) maximum value of FFR gradient over 20 mm; and 2) length of functional disease, which was defined as the distance between the points with FFR drop ≥0.0015/mm. Using these acquired values, the pullback pressure gradient (PPG) index was defined as a ratio between maximum FFR gradient over 20 mm and FFR gradient in total vessel (1 − distal FFR), modified by relative length of functional disease to total vessel length. Thus, PPG index integrates the relative contribution of both focal FFR step-up in the vessel and its length. The PPG index is a continuous metric: values approaching 1.0 represent hemodynamically focal coronary artery disease, whereas values close to 0 represent diffuse disease. The patterns of coronary artery disease were classified into focal (highest tertile of PPG index), diffuse (lowest tertile), and mixed (intermediate tertile). In comparison with angiographic assessment of disease patterns, which was made subjectively by 2 independent researchers, 53% of the cases showed discordant classification. There was no significant difference in MACE rates according to classification by PPG index in 40 patients with median follow-up of 122 days. Although this study provided a novel index that can objectively discriminate physiological focal versus diffuse disease, insufficient clinical outcome data according to different disease patterns warrant further validation of this concept.

Lee et al. (19) evaluated an alternative method of instantaneous FFR gradient per unit time (*dFFR(t)/dt*) using pre-PCI manual FFR pullback curves from 516 patients (Figure 6C). The *dFFR(t)/dt* is a continuous metric, and higher values represent higher instantaneous FFR gradients across the target stenosis and higher expected gain from focal treatment. Based on previous studies evaluating the relationship between trans-stenotic pressure gradient and FFR (68,75), coronary stenosis with trans-stenotic pressure gradient >15 mm Hg was defined as a major FFR gradient lesion and corresponding cut-off value of *dFFR(t)/dt* was ≥0.035/s. Conversely, *dFFR(t)/dt* <0.015/s represented vessels with no atherosclerotic involvement on IVUS and trans-stenotic pressure gradient <5 mm Hg. Any stenosis with *dFFR(t)/dt* of 0.015 to 0.034/s was considered a minor FFR gradient lesion. According to the presence of major and/or minor FFR gradient lesions, the disease patterns were classified into major (physiologically focal disease), minor (physiologically diffuse), or mixed FFR gradient groups. In validation cohorts in which all patients underwent PCI based on pre-PCI FFR ≤0.80, the major FFR gradient group showed the lowest probability of suboptimal post-PCI physiological results (10%). Conversely, the minor FFR group showed the lowest probability of optimal post-PCI physiological results (26.1%). At 2 years, patients with suboptimal post-PCI physiological results showed a significantly higher risk of TVF than those with optimal post-PCI physiological results (HR: 4.322; 95% CI: 1.265 to 14.767; p = 0.019). This study demonstrates that characterization of coronary atherosclerotic disease can be feasible using manual FFR pullback in the pre-PCI phase, and that the novel index of *dFFR(t)/dt* provides information about the probability of optimal post-PCI physiological results from stenting for the individual stenosis. Further study is warranted to support routine use of these approaches in daily practice.

## Areas of Uncertainty and Future Directions

### Functionally-optimized PCI versus optimal medical treatment alone

The ISCHEMIA (International Study of Comparative Health Effectiveness with Medical and Invasive Approaches) trial has re-emphasized the value of optimal medical treatment in patients with SIHD (19). However, the use of physiology-guided revascularization in the ISCHEMIA trial was relatively low (20.3% of initial invasive strategy arm and 9.3% of the total trial population). Because part of the relative superiority of optimal medical treatment resulted from higher periprocedural myocardial infarction rates in the revascularization arm, better planning and guidance of PCI with physiological assessment might have resulted in lower rates of that endpoint. This hypothesis is supported by the marked reduction in periprocedural myocardial infarction noted in SYNTAX II, a physiology-based revascularization trial, compared with the original SYNTAX study in which revascularization was mostly performed by angiographic planning (20). Furthermore, it should be noted that no previous study, including the ISCHEMIA trial, evaluated comparative prognosis between functionally optimized revascularization and optimal medical treatment. As discussed in the previous text, angiographically successful PCI does not necessarily result in functionally optimized PCI, which was consistently shown to have significantly better prognosis than functionally suboptimal PCI. Whether “ischemia-resolving PCI” would enhance patient prognosis still needs further clarification through future trials.

### Procedural planning for functionally-optimized PCI without pressure wire

Physiological characterization of coronary atherosclerotic disease using pressure wire pullback tracings enables selection of optimal revascularization target and procedural planning to raise the probability of acquiring optimal post-PCI physiological results. However, accurate pressure wire pullback maneuvers can be challenging. Conversely, simulation or image-based computational FFR methods, such as computed tomography–derived FFR (FFR_CT_) or angiography-derived FFR, can easily reproduce FFR step-up patterns, virtually modify the stenosis, and thus provide predicted post-PCI results (75). Therefore, procedural planning with these methods is potentially more convenient and applicable in daily practice. This concept is still under investigation, with a currently ongoing P3 (Precise Percutaneous Coronary Intervention Plan Study) (NCT03782688) study, a multicenter prospective registry evaluating the accuracy of FFR_CT_ planner with invasive post-PCI FFR as a reference. This study will better clarify the clinical implications of pressure-wire–free procedural planning.

### Currently ongoing randomized controlled trials evaluating clinical implications of physiology-based pre-procedural planning

There are a number of studies currently evaluating clinical relevance of physiology-based pre-procedural planning. In addition to the DEFINE-GPS study, which will compare TVF at 2 years between iFR pullback and iFR-angiography coregistration system-based PCI versus angiography-guided PCI (NCT04451044), PICIO (Pullback wIth Resting Full-Cycle Flow ratIO or Fractional Flow Reserve for the Prediction of Post-PCI Hemodynamic Outcomes) (NCT04417634) study will compare the capacity of pre-procedural resting full-cycle ratio gradients versus FFR gradients to predict the hemodynamic outcomes after PCI.

Pre-procedural planning without pressure wire using FFR_CT_ planner (P3 study, NCT03782688) or angiography-derived FFR is also under evaluation. AQVA (Angio-based Quantitative Flow Ratio Virtual PCI Versus Conventional Angio-guided PCI in the Achievement of an Optimal Post-PCI QFR) (NCT04664140) is an ongoing randomized clinical trial evaluating efficacy of pre-procedural planning based on quantitative flow ratio, compared with angiography-guided PCI. These studies will further clarify the clinical impact of pre-procedural planning on post-PCI physiological results and clinical outcome.

## Conclusions

The purpose of revascularization is not just to alleviate angiographic stenosis but to resolve myocardial ischemia and improve prognosis. Post-PCI physiological assessment with FFR or NHPRs provides information about the functional results of revascularization and prognostic stratification after PCI. In suboptimal post-PCI physiological results, further investigations and interventions aimed to find potential reasons can improve the final result and patient prognosis. Both post-PCI value and physiological gain from PCI are important to further stratify patients with higher risk of future clinical events. Recent evidence supports the importance of pre-PCI pullback analysis to characterize the patterns of coronary atherosclerotic disease to better identify the optimal revascularization target, simplify the procedure, and raise the probability of optimal post-PCI physiological results. Physiological assessments by FFR or NHPRs are practical and applicable tools enabling not just determination of revascularization but also functional optimization of PCI to improve outcomes (Central Illustration). Whether procedural planning guided by pre-PCI pullback analysis or further intervention guided by post-PCI physiological assessment will lead to improved clinical outcomes awaits the results of randomized clinical trials.

**Central Illustration:**
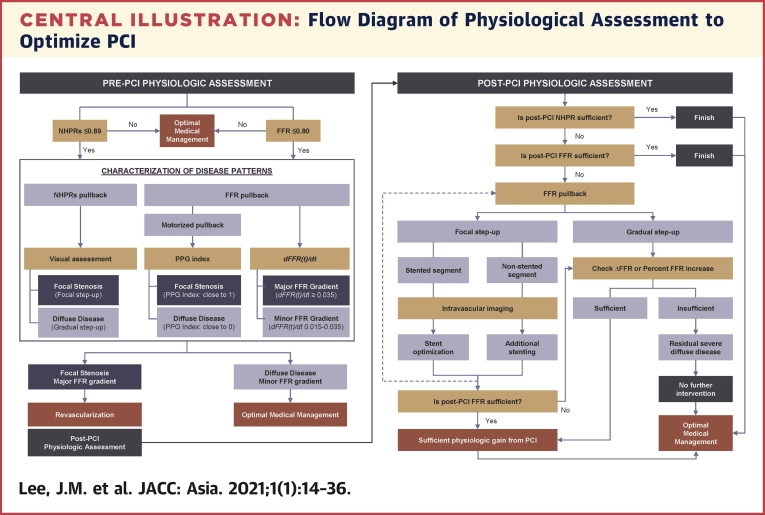
Flow Diagram of Physiological Assessment to Optimize PCI Pre-PCI physiological assessment is summarized **(left panel)**. After confirming the presence of vessel-related myocardial ischemia by FFR or NHPRs, physiological disease patterns can be characterized by pullback curve analysis. The NHPR pullback curve is interpreted based on visual assessment. PPG index can numerically quantify the disease pattern. *dFFR(t)/dt* is a simplified method to clarify the disease pattern and predict the optimal physiological results after PCI. Regardless of methodology, focal disease (or major FFR gradient) is the optimal target of revascularized, whereas diffuse disease (or minor FFR gradient) might not be the optimal target for local treatment with stent implantation. After revascularization, post-PCI physiological assessment is recommended to functionally optimize the results **(right panel)**. In satisfactory post-PCI NHPRs, procedure can be finished without additional evaluation. However, if the post-PCI NHPRs are not sufficient, the measurement of FFR is recommended. When the post-PCI FFR is not sufficient, FFR pullback curve analysis and intravascular imaging would be helpful to evaluate the cause of suboptimal physiologic results. When the final physiologic result is not sufficient with gradual step-up pattern, FFR gain from PCI would provide further stratification of patients with residual diffuse disease in which additional procedures would not improve the final physiological results. *dFFR(t)/dt =* instantaneous fractional flow reserve gradient per unit time; FFR = fractional flow reserve; NHPRs = nonhyperemic pressure ratios; PCI = percutaneous coronary intervention; PPG = pullback pressure gradient.

## Funding Support and Author Disclosures

Dr. Joo Myung Lee has received a research grant from Abbott Vascular and Philips Volcano; and has received consulting fees from RainMed. Dr. Matsuo has received institutional research support by Phillips; and has received consulting fees from Zeon Medical. Dr. Koo has received an institutional research grant from Abbott Vascular and Philips Volcano. Dr. Fearon has received institutional research support from Abbott Vascular, Medtronic, and Edwards Lifesciences; and has been a consultant with CathWorks and with HeartFlow. All other authors have reported that they have no relationships relevant to the contents of this paper to disclose.
